# Effect evaluation of different preventive measures for ileus after abdominal operation: A systematic review and network meta-analysis

**DOI:** 10.1016/j.heliyon.2024.e25412

**Published:** 2024-02-02

**Authors:** Yan Cui, Chengzu Zhang, Hui Zhang, Xuan Zhang, Yuan Tang, Zhihang Wu, Tianming Wang, Quanxin Chen, Ying Meng, Bo Wang, Mei Liu, Jianfeng Yi, Yuhong Shi, Richeng Li, Haibang Pan

**Affiliations:** a First School of Clinical Medicine, Gansu University of Chinese Medicine, Lanzhou, Gansu, China; b Department of Pharmacy, Expo High-tech Hospital, Zibo, Shandong, China; c School of Nursing, Gansu University of Chinese Medicine, Lanzhou, Gansu, China; d Key Laboratory of Gansu Provincial Prescription Mining and Innovative Translational Laboratory, Gansu University of Chinese Medicine, Lanzhou, 730000, Gansu, China; e Gansu Provincial Traditional Chinese Medicine New Product Creation Engineering Laboratory, Gansu University of Chinese Medicine, Lanzhou, 730000, Gansu, China

**Keywords:** Abdominal surgery, Ileus, Prevention, Network meta-analysis

## Abstract

**Background:**

Different approaches to the prevention of postoperative ileus have been evaluated in numerous randomized controlled trials. This network meta-analysis aimed to investigate the relative effectiveness of different interventions in preventing postoperative ileus.

**Methods:**

Randomized controlled trials (RCTS) on the prevention of postoperative ileus were screened from Chinese and foreign medical databases and compared. STATA software was used for network meta-analysis using the frequency method. Random-effects network meta-analysis was also used to compare all schemes directly and indirectly.

**Results:**

A total of 105 randomized controlled trials with 18,840 participants were included in this report. The results of the network meta-analysis showed that intravenous analgesia was most effective in preventing the incidence of postoperative ileus, the surface under the cumulative ranking curve (SUCRA) is 90.5. The most effective intervention for reducing the first postoperative exhaust time was postoperative abdominal mechanical massage (SUCRA: 97.3), and the most effective intervention for reducing the first postoperative defecation time was high-dose opioid antagonists (SUCRA: 84.3). Additionally, the most effective intervention for reducing the time to initiate a normal diet after surgery was accelerated rehabilitation (SUCRA: 85.4). A comprehensive analysis demonstrated the effectiveness and prominence of oral opioid antagonists and electroacupuncture (EA) combined with gum.

**Conclusion:**

This network meta-analysis determined that oral opioid antagonists and EA combined with chewing gum are the most effective treatments and optimal interventions for reducing the incidence of postoperative ileus. However, methods such as abdominal mechanical massage and coffee require further high-quality research.

## 1 Introduction

Postoperative ileus (POI), a concerning complication following surgery, continues to play a significant role in patient prognosis [1]. It is characterized by temporary inhibition of gastrointestinal peristalsis and prolonging the time for patients to begin to exhaust and defecate. Complex pharmacological (opioid and anesthetic), neurological, and immune-mediated pathways play a role in its pathogenesis [2]. Despite extensive research focusing on the high-risk population, 10 %–30 % of patients still experience POI after abdominal surgery, potentially due to the traumatic nature of the procedure [1]. Patients who experience different levels of exposure over time lose their peritoneal integrity, resulting in extended hospital stays, increased financial burden, and symptoms related to ileus, such as prolonged exhaustion and defecation times, abdominal distension, abdominal pain, nausea, and vomiting. Although postoperative ileus is typically inevitable, experimental investigations have sought to identify approaches that can reduce its duration and facilitate the restoration of gastrointestinal function soon after surgery [[3], [4], [5]]. Drug and non-drug therapies are primarily used to prevent and cure postoperative gastrointestinal dysfunction (POGD) [6], and prevention is prioritized over treatment for better recovery after surgery. Currently, pharmacological therapies such as opioid receptor antagonists, intravenous analgesia, epidural analgesia, and conventional herbal decoctions are the mainstay of treatment for intestinal blockage [7]. Acupuncture, early diet, fake feeding, stomach massage, and other techniques are examples of non-drug therapies [8,9]. It is currently unclear which approach is the most effective for preventing postoperative intestinal blockage following abdominal surgery, as a significant number of Randomized controlled trials (RCTS) investigations have shown inconsistent experimental results. To offer suggestions for the clinical diagnosis and treatment of postoperative ileus and to identify a more general prevention strategy for clinical practice, the author of this research article screened randomized controlled trials and evaluated the best intervention measures to prevent postoperative ileus after abdominal operation through a network meta-analysis and systematic review.

## 2 Methods

### 2.1 Search strategy

A thorough search for related research was undertaken across many databases, including Chinese data from the CNKI, VIP, WANFANG, and CBM databases, and English data from the Cochrane Library, Embase, Web of Science, and PubMed databases. The search terms included prevent, postoperative ileus, ileus, and free words. The search duration is not restricted, and languages such as Chinese and English are available. In addition, references in the initial meta-analyses and review articles were searched for and reviewed.

### 2.2 Selection of research studies

This study followed the PRISMA guidelines. The inclusion and exclusion of studies for the focused questions mentioned above were guided by the Population, Intervention, Comparison, and Outcomes (PICO) framework:

Population (P): patients who had undergone abdominal surgery without restrictions on age or sex.

Intervention (I): prophylactic administration refers to any therapies that are intended to avoid postoperative ileus.

Comparison (C): every conceivable comparison between the therapies that were included was thoroughly examined.

Outcome (O): the occurrence of postoperative ileus, the duration until the first bowel movement, the duration until the first defecation, and the duration until resuming a regular diet.

Inclusion Criteria.

• Patients undergoing abdominal surgery.
• randomized controlled trial (RCT).
• The clinical outcomes evaluated and documented throughout the follow-up period included the occurrence of ileus, duration until the first expulsion of gas, duration until the first bowel movement, and duration until resumption of a regular diet.

Exclusion Criteria.

• Non-abdominal surgical procedures, administration without preventive intent, animal trials, protocols, absence of a control group, examination, anecdotal accounts, unavailability of data or complete text, and statistical investigation is impractical.

### 2.3 Data extraction

Two researchers reviewed all the titles and abstracts of the retrieved articles. After the preliminary exclusion and inclusion of the literature, the full texts were downloaded for further evaluation. Any discrepancies between the two reviewers were resolved through consultation with a third researcher to establish the final inclusion criteria. The basic information of the included studies was as follows: first author name, publication date, country, type of surgery, sample size, mean age, interventions, and outcome indicators.

### 2.4 Quality assessment

The Cochrane Collaboration tool was used to assess the risk of bias in randomized controlled trials (RCTs), which consisted of random sequence generation, allocation concealment, participant and personnel blinding, outcome assessment blinding, incomplete outcome data, selective reporting, and other biases. Visualization was achieved using the RevMan software (Version 5.4.1; The Cochrane Collaboration, 2020), as presented in the evaluation results in Fig. 2.

### 2.5 Statistical analysis

Network meta-analyses are statistical studies that examine more than two treatments by using indirect comparisons or a combination of indirect and direct comparisons. Network meta-analysis, in comparison to classic meta-analysis, has the ability to identify the most effective intervention among several options. Stata software (version 15; STATA Corporation, College Station, TX, USA) was used to draw network evidence maps of different interventions to visually represent the evidence quantity and compare the relationships between different interventions. First, direct comparisons between different interventions were performed. A network meta-analysis was performed using the frequentist framework random-effects model, allowing indirect and direct evidence to be combined into the analysis, and the efficacy of different interventions to be ranked. The inconsistency test was first performed when the test results were P > 0.05, which implied no statistical significance and indicated consistency, and a consistency model was employed for the network meta-analysis. The Surface Under the Cumulative Ranking Curve (SUCRA) measure was used to evaluate and rank the efficacy of each therapy, ultimately determining the most successful treatment. Finally, a funnel plot was constructed to present the publication bias for all available treatments. Statistical significance was set at P < 0.05 for all measurements.

## 3 Result

### 3.1 Literature screening and basic data information

**Fig. 1:**
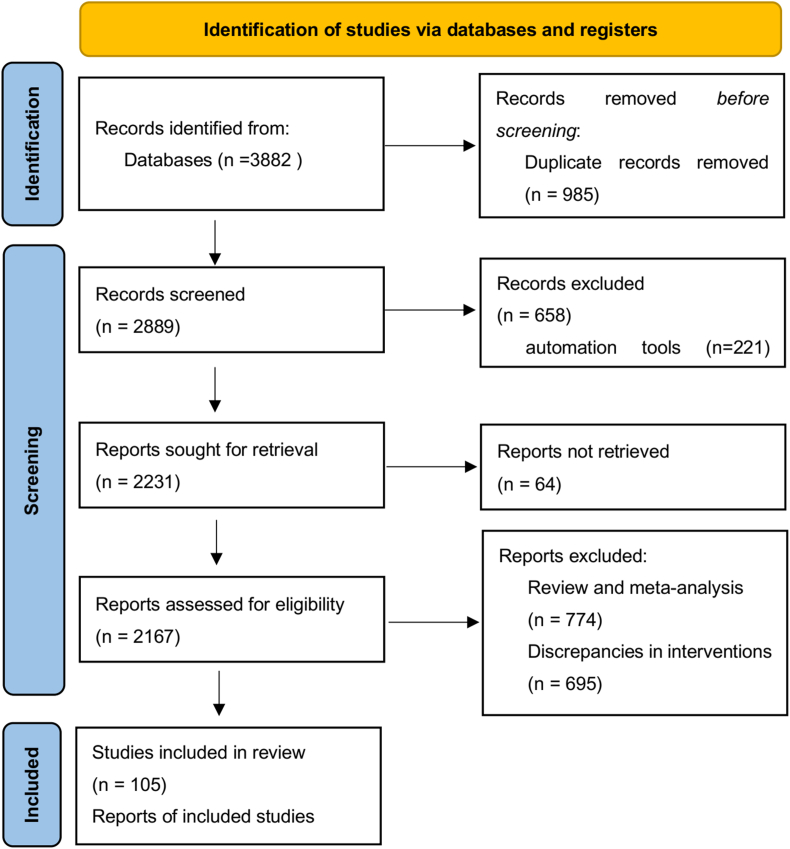
Flow chart of data retrieval and screening.

A total of 18,840 participants and 30 different types of therapies were included in 105 randomized controlled trials [[10], [11], [12], [13], [14], [15], [16], [17], [18], [19], [20], [21], [22], [23], [24], [25], [26], [27], [28], [29], [30], [31], [32], [33], [34], [35], [36], [37], [38], [39], [40], [41], [42], [43], [44], [45], [46], [47], [48], [49], [50], [51], [52], [53], [54], [55], [56], [57], [58], [59], [60], [61], [62], [63], [64], [65], [66], [67], [68], [69], [70], [71], [72], [73], [74], [75], [76], [77], [78], [79], [80], [81], [82], [83], [84], [85], [86], [87], [88], [89], [90], [91], [92], [93], [94], [95], [96], [97], [98], [99], [100], [101], [102], [103], [104], [105], [106], [107], [108], [109], [110], [111], [112], [113], [114]], which were obtained from various databases. The essential details of these trials are presented in (Supplementary Table S1). Two treatment categories were based on different doses of the same class of medication. Routine postoperative care served as the intervention in the control group. However, the nursing strategy varied depending on the surgical technique used. In Fig. 1, the retrieval procedure and outcomes are displayed.

**Fig. 2:**
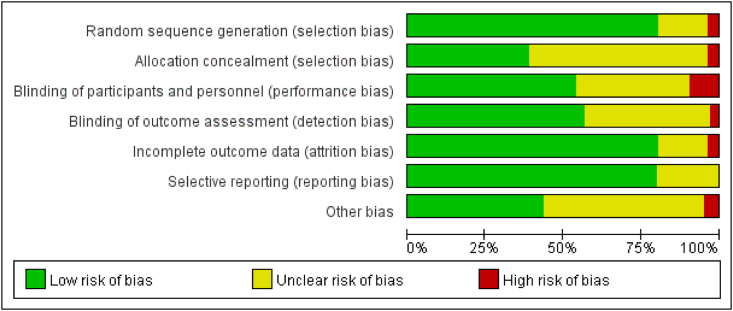
Figure of risk of bias assessment.

All 105 studies included in the study were randomized controlled trials, and 85 of these studies provided a detailed explanation of the randomization process. Blinding research participants to acupuncture and moxibustion therapies is challenging owing to the nature of these treatments. The lack of a clear explanation and potential for follow-up bias makes it a high-risk procedure. Fig. 2 shows the risk of bias assessment, with the opinions of the review authors on each risk of bias item presented as a percentage of all included studies.

**Fig. 3:**
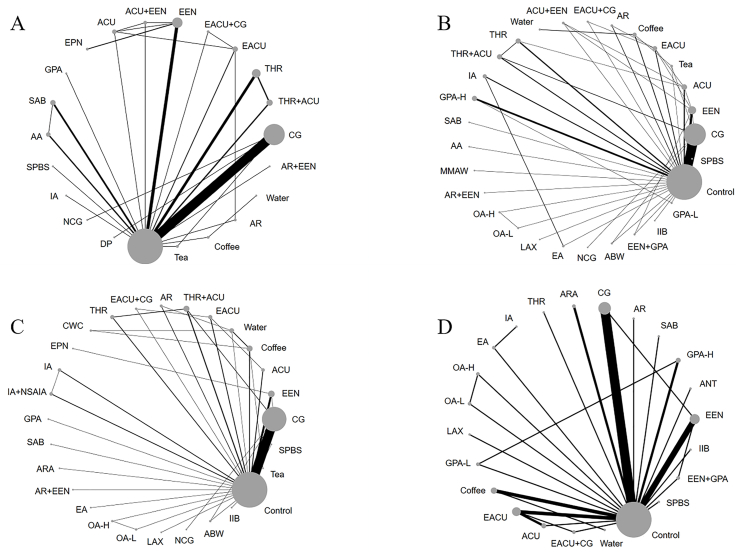
Prevention of all interventions network maps after postoperative ileus. Figure A: The incidence of postoperative ileus; Figure B: The first exhaust time; Figure C: The first time of bowel movement; Figure D: The normal eating time. CG: Chewing gum; THR: Traditional herbal remedies; ACU: Acupuncture; EACU: Electroacupuncture; EEN: Early enteral nutrition; EPN: Early parenteral nutrition; GPA: Gastric prokinetic agents; SAB: Seprafilm Adhesion Barrier; AA: Antiadhesive agent; SPBS: Stimulation with Probiotics before Surgery; IA: Intravenous analgesia; NCG: Nicotine chewing gum; DP: Dermal patch; MMAW: Mechanical Massage of the Abdominal Wall; IOA: Infusion of local anesthetic; AR: Accelerated rehabilitation; OA: Opioid antagonist; LAX: Laxative; ABW: Acupressure bracelet worn; IIB: Intestinal isolation bag; CWC: Coffee without caffeine; ARA: Adrenergic receptor agonists; NSAIA: Non-steroidal anti-inflammatory agents; EA: Epidural analgesia; ANT: Antemetic.

The primary prognostic indicator was the incidence of postoperative intestinal blockage, with the first occurrence of exhaustion, the first occurrence of bowel movement, and normal eating time serving as supplementary outcome indicators. Fig. 3 shows the primary outcome indicators, postoperative ileus intervention measures, and secondary end-index network diagram.

### 3.2 The incidence of postoperative ileus

**Fig. 4:**
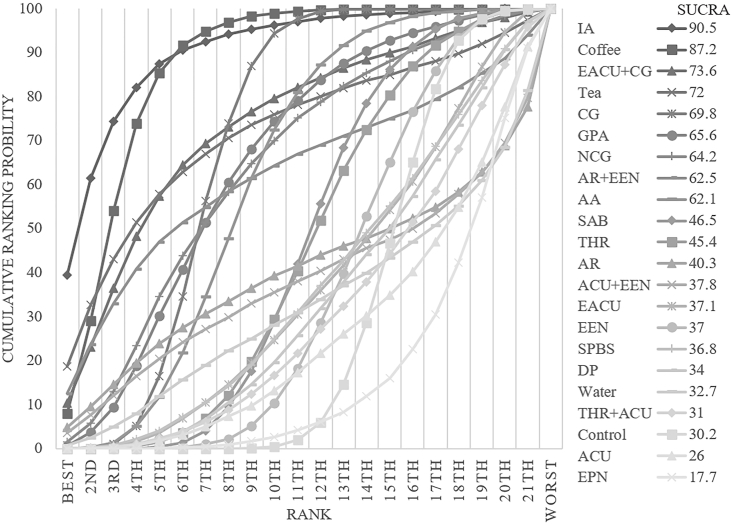
Ranking of cumulative probability of bowel obstruction reduction for each intervention measured by incidence of postoperative bowel obstruction.

The consistency model was used for analysis after conducting an inconsistency test, which resulted in a p-value of 0.8544. The inconsistency test was not significant. A comparison network of several therapies employed to prevent postoperative ileus is shown in Fig. 3A. Among these interventions, IA was the first substantially effective intervention, reducing the incidence of ileus by 2.56 % compared to the control group (95 % CI [−4.79,-0.33]). Supplementary Table 2 (S2) compares the interventions. With a cumulative ranking probability of 90.5, IA was the most effective strategy for reducing the risk of postoperative ileus (Fig. 4).

### 3.3 The first exhaust time

**Fig. 5:**
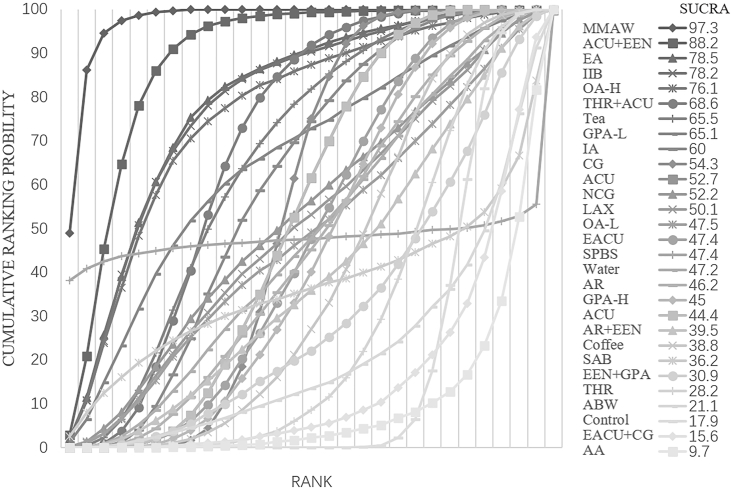
Ranking of cumulative probability of a reduction in ileus by each intervention measured as time to first flatus.

The consistency model was used for analysis after conducting an inconsistency test, which resulted in a p-value of 0.4297. The inconsistency test was not significant. A comparison network of several therapies to reduce the first exhaust time is shown in Fig. 3B. MMAW was the first substantially effective intervention, reducing exhaustion time by 43.20 h on average compared to the control group and by 95 % CI (−61.91, −24.48). For a comparison of the interventions, see Supplementary Table 3 (S3). With a cumulative ranking probability of 97.3, MMAW was the most effective strategy for reducing the first exhaust time after surgery (Fig. 5).

### 3.4 The first time of bowel movement

**Fig. 6:**
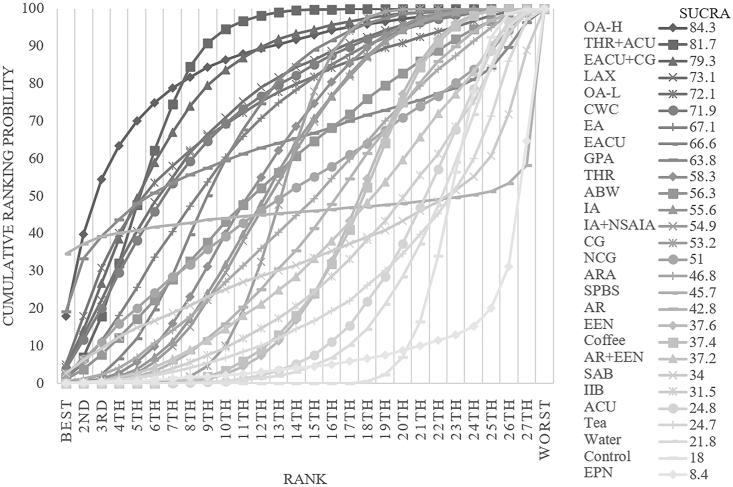
Ranking of cumulative probability of a reduction in ileus by each intervention measured as time to first bowel movement.

The consistency model was used for analysis after conducting an inconsistency test, which resulted in a p-value of 0.4297. The inconsistency test was not significant. A comparison network of several therapies used to reduce the first bowel movement time is shown in Fig. 3C. OA-H was the first substantially effective intervention, reducing exhaustion time by 41.00 h on average compared to the control group and by 95 % CI (−79.36, −2.64). For a comparison of the interventions, see Supplementary Table 4 (S4). With a cumulative ranking probability of 84.3, OA-H was the most effective strategy to reduce the first exhaust time after surgery (Fig. 6).

### 3.5 The return to normal eating time

**Fig. 7:**
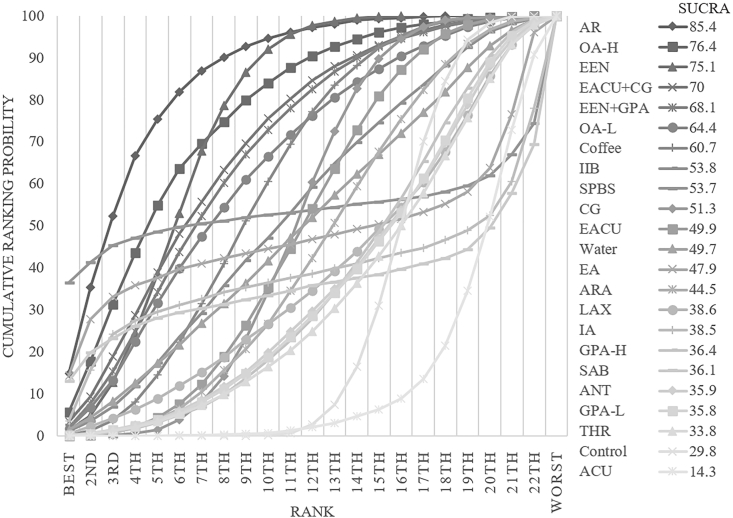
Ranking of cumulative probability of a reduction in ileus by each intervention measured as time to normal eating.

The consistency model was used for analysis after conducting an inconsistency test, which resulted in a p-value of 0.6685. The inconsistency test was not significant. A comparison network of several therapies for reducing the return to normal eating times is shown in Fig. 3D. AR was the first substantially effective intervention, reducing the time to return to normal eating time by 43.00 h on average compared to the control group and by 95 % CI (−76.52, −9.49). For a comparison of the interventions, see Supplementary Table 5 (S5). With a cumulative ranking probability of 85.4, AR was the most effective strategy for reducing the time to return to normal eating after surgery (Fig. 7).

The best intervention among the top five cumulative probabilities of the four outcomes was screened.

**Table 1:** Summary of the cumulative probability rankings of the top-ranked interventions and EACU + CG for each outcome measure.

	IA	MMAW	OA-H	AR	EACU + CG
The incidence of postoperative ileus	90.5*	97.3*	–	40.3	73.6^#^
The first exhaust time	60	–	76.1^#^	46.2	15.6
The first time of bowel movement	55.6	–	84.3*	42.8	79.3^#^
The normal eating time	38.5	–	76.4^#^	85.4*	70^#^

“*“: The cumulative probability of each index ranked first; “#“: The cumulative probability of each index ranked the top 5.

**Fig. 8:**
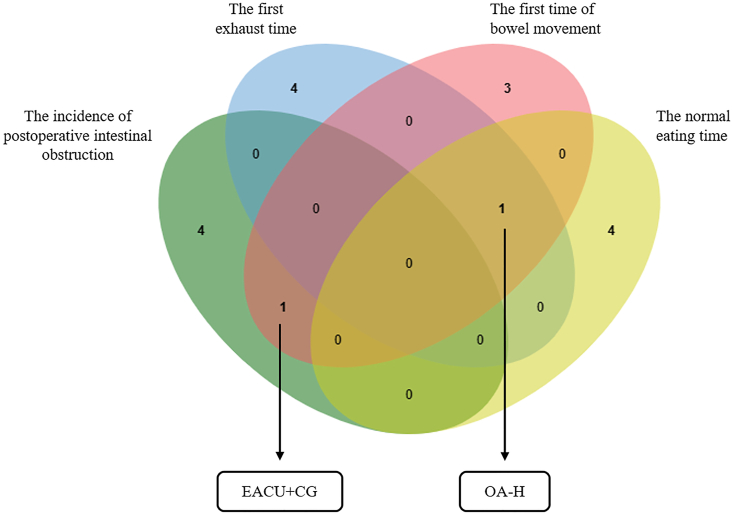
Venn diagram of the top 5 interventions in the prevention of postoperative ileus in different outcome indicators.

Fig. 8 The interventions with good results in different outcome measures, OA-H and EACU + CG, were obtained through a Venn diagram. Table 1 presents the cumulative probabilities of the most effective and the combined best effective interventions for the four outcome measures, respectively.

## 4 Discussion

According to the findings of our network meta-analysis, the OA-H and EACU + CG had a positive effect on restoring postoperative intestinal recovery and preventing ileus, which was essentially consistent with the current clinical medication regimen for the prevention of postoperative ileus. Opioid analgesics are routinely administered during and after surgery to treat intraoperative and postoperative pain [115]. In addition to their cerebral effects on pain receptors, they also have negative GI effects, such as reduced gastric motility and emptying, suppression of intestinal propulsion, and altered fluid and electrolyte balance, which can lead to intestinal blockage [116]. The only licensed drugs in this class are methylnaltrexone (MNTX) and alvimopan, which selectively eliminate or attenuate peripheral opioid-induced side effects without affecting the central analgesic effect, owing to their polarity, large structure, and low lipid solubility, which prevent them from crossing the blood-brain barrier [117]. The peripherally-acting mu-opioid receptor antagonist (PAMORA) activity was restricted to the periphery. According to Tyler's meta-analysis, the use of selective opioid antagonists reduced the incidence of POI in patients undergoing bowel resection and minimized hospitalization expenditure for patients undergoing intestinal resection [118]. The drug used in the clinical trials in the included studies was alvimopan [94]; the total dosage of alvimopan was restricted to 15 doses, with instructions to take a 12-mg capsule 30 min to 5 h before surgery and a 12-mg capsule twice daily from the day of the operation until discharge [116], for up to seven days. GI2 was an objective composite endpoint assessed based on the time the patient first tolerated solid food (a sign of upper GI tract function recovery) and the time when the patient first defecated (a measure of lower GI tract function recovery). The time to the GI2 endpoint was shortened in the alvimopan group compared with the placebo group in most trials, regardless of whether it was a main or secondary endpoint, similar to the results of the network meta-analysis of the current investigation. Sameh et al. investigated many therapeutic approaches targeting the reduction of postoperative ileus incidence and severity after colorectal surgery. These results are consistent with our own studies, suggesting that avimopan is a common and successful method for reducing intestinal obstruction after surgery for colorectal cancer [119]. Electroacupuncture, in conjunction with chewing gum, is identified as an effective prophylactic therapy for postoperative ileus in this research. While this combination is not universally used, it is extensively utilized in China and other Asian nations, such as Korea. Acupoint stimulation, represented by acupuncture, has been used for thousands of years in China and has gradually become an important supplement to the comprehensive prevention and treatment strategy for POGD owing to its advantages of multi-target, precise effect, and no toxic side effects. Transcutaneous electrical acupoint stimulation (TEAS), a mature modern acupoint stimulation method, combines transcutaneous electrical nerve stimulation and acupoint stimulation. It is simpler, more convenient, noninvasive, and has the same therapeutic effect as traditional hand acupuncture [6]. This is more acceptable to patients. Multiple studies have shown that EACU may effectively prevent and cure postoperative ileus by skillfully managing local inflammation. This is accomplished by suppressing the activity of inflammatory molecules, such as TNF-α, IL-6 [120], and JAK2/STAT3 [121] inflammatory signaling pathways, in the gastrointestinal tract and peritoneal cavity. Furthermore, EACU has the capability to modify the movement of the small intestine and the process of emptying the stomach, perhaps due to its ability to increase levels of ghrelin and motilin, as well as the activity of the autonomic nervous system, particularly the vagus nerve [122,123]. Additionally, it may alleviate pain after surgery by influencing cytokines [124].

Gum chewing is a non-pharmacological, affordable, well-tolerated, secure, and efficient method for reducing intestinal blockage following colorectal surgery [125]. The mechanism of action is similar to that of early postoperative feeding [126]. Research has shown that chewing gum increases the levels of ghrelin via the act of chewing or the production of gastrin [81]. Furthermore, engaging in oral stimulation and chewing may activate the vagus nerve, which plays a role in facilitating peristalsis [69]. The efficacy of chewing gum in avoiding postoperative paralytic ileus is still inconclusive due to contradictory findings. For instance, a research shown that chewing gum is linked to the prompt restoration of gastrointestinal function after gynecological cancer surgery. It may serve as an efficient and safe measure to avoid postoperative ileus [127]. In contrast, a separate research indicates that gum chewing does not have a correlation with accelerated gastrointestinal healing after surgery in children [128]. This discrepancy may arise from variations in outcomes attributable to diverse research cohorts. The objective of this research was to examine the overall efficacy of various preventative interventions in all abdominal operations. Postoperative gum chewing was placed in the middle of the range of interventions in our network analysis, lowering the incidence of bowel obstruction prevention and the time to the first bowel movement and flatus, but it had no impact on the time to return to a regular diet. Furthermore, despite the NCG ranking second, there was no distinction between the NCG and control groups. Chewing gum and electroacupuncture ranked in the top five in the prevention of postoperative intestinal blockage, first feces time, and time to resume a regular diet, suggesting that this combination may be a useful and affordable preventative strategy.

While there are numerous common meta-analyses, there are few network meta-analyses on the prevention of postoperative intestinal blockages. Our analysis revealed that early diet consistently ranked in the middle of the four outcome indicators. A previous network meta-analysis revealed that early diet and epidural anesthesia were the most effective interventions for the prevention of postoperative ileus following colorectal surgery [129]. However, the findings of the pairwise comparison revealed that there was no difference in the other three indicators, and that only the time it took to resume a regular diet was superior to that of the control group. Postoperative coffee consumption following colorectal and gynecological surgery can lower the POI and LOS [130], according to a meta-analysis. In this study's analysis, coffee ranked second in preventing postoperative ileus and outperformed EEN, SPBS, DP, THR + ACU, control, ACU, and EPN. However, it did not promote exhaustion or feces, although it may accelerate the iniatiation of a regular diet. Improved recovery programs have evolved into industrial standards of care in several surgical specialties. According to a meta-analysis of gynecological transabdominal surgery, improved recovery successfully reduced the length of hospital stay without increasing the risk of ileus [131]. Our findings demonstrate that AR was superior to other therapies in shortening the interval before normal eating, although the difference was not statistically significant.

## 5 Limitations

All the RCTS included in this study had high evidence credibility, but there were also some limitations. The included procedures included both laparoscopic and open procedures, and studies have shown that laparoscopic procedures are associated with a reduced risk of postoperative ileus during and after gastric surgery [132]. The following research might individually examine endoscopic surgery and open surgery to ascertain the benefits and drawbacks of various treatments performed via endoscopic or open surgical procedures. Second, differences in the doses of some drugs within the same class were ignored, which may have biased the results, investigating the dose-correlation of various medicines in preventing postoperative ileus is another promising area of inquiry. In addition, to reduce heterogeneity, we excluded studies with a sample size of less than 30, which may have prevented the interventions we included from creating more closed loops that could allow direct comparisons and reduce confidence.

## 6 Conclusion

Adhesions are a frequent complication after abdominal surgery. However, how to avoid it is debatable. According to the findings of this network meta-analysis, alvimopan and non-pharmacological electroacupuncture combined with chewing gum may be the most effective strategies for enhancing bowel function recovery and minimizing the incidence of intestinal blockage following abdominal surgery. The recommended dose for alvimopan is one 12 mg capsule taken 30 min to 5 h before surgery, and another 12 mg capsule taken twice daily from the first postoperative day until discharge, for a maximum of 7 days. Therapeutic doses of alvimopan should not be used for more than 7 days prior to starting opioids. Electroacupuncture was applied to Neiguan (PC6), the currently recognized standard acupoint for the prevention of post operative nausea and vomiting (PONV); however, the optimal time of acupoint stimulation intervention is controversial in the academic community, and there is no unified standard. Chewing gum 2 h after the surgery, one or two pieces of sugar-free gum were chewed every 2 h for 15 min each time, with a 2-h interval between the chewing, until anal exhaust. Further, the chewing of the gum was stopped at night. These two interventions, as identified by the network meta-analysis, have their own advantages and disadvantages. However, the literature support for these interventions is limited, and further high-quality RCTs are needed to confirm our results.

## Funding statement

This study received funding from the 10.13039/501100001809National Natural Science Foundation of China (81860850) , Gansu Education Department (2023A-076), and Lanzhou City Science and Technology Bureau (2020-XG-26)

## Additional information

No additional information is available for this paper.

## Data availability statement

The data that support the findings of this study are available from the corresponding author, [HBP], upon reasonable request.

## Ethics declarations

Review and/or approval by an ethics committee was not needed for this study because [This work is a review of the literature and does not address the ethical considerations of animal, cell, and human experimentation.].

## CRediT authorship contribution statement

**Yan Cui:** Writing – original draft, Writing – review & editing. **Mei Liu:** Data curation. **Hui Zhang:** Data curation, Formal analysis. **Xuan Zhang:** Formal analysis, Methodology. **Yuan Tang:** Methodology, Software. **Zhi Hang Wu:** Visualization. **Ri Cheng Li:** Conceptualization. **Quan Xin Chen:** Writing – review & editing. **Ying Meng:** Conceptualization, Formal analysis. **Bo Wang:** Project administration, Supervision. **Hai Bang Pan:** Funding acquisition, Project administration, Supervision, Writing – review & editing.

## Declaration of competing interest

The authors declare the following financial interests/personal relationships which may be considered as potential competing interests: Haibang Pan reports article publishing charges was provided by Haibang.
