# Supplementary material for: Effect evaluation of different preventive measures for ileus after abdominal operation: A systematic review and network meta-analysis
Source: Heliyon. 2024 Feb 2;10(4):e25412. doi: 10.1016/j.heliyon.2024.e25412 (PMC10867618; doi:10.1016/j.heliyon.2024.e25412)
Supplement: Multimedia component 1 [file mmc1.docx]

**Appendices**

S1 The basic information of the literature was included

| ID | Study | Year | Type of surgery | State | Intervening Measure | | sample size | | Age(x±s) | | Outcome |
| --- | --- | --- | --- | --- | --- | --- | --- | --- | --- | --- | --- |
|  |  |  |  |  | Group1 | Group2 | Group1 | Group2 | Group1 | Group2 |  |
| 1 | Qiu Fan | 2006 | Gynecologic abdominal surgery | CHINA | Chewing Gum | Control | 64 | 64 | 52.09 | 50.86 | A, B |
| 2 | I. J. M. Han-Geurts | 2007 | Open colorectal or abdominal vascular surgery | Netherlands | Early Enteral Nutrition | Control | 61 | 67 | 63(18–82) | 67(33–87) | A, B, C, D |
| 3 | Song Jianqiao | 2009 | Abdominal operation | CHINA | Acupuncture | Control | 90 | 90 | 53.8 | 54.5 | A, C |
| 4 | Zhong Zifeng | 2009 | Radical resection of rectal cancer | CHINA | Chewing Gum | Control | 60 | 60 | NR | NR | A, C |
|  |  |  |  |  | Tea |  | 60 |  | NR |  |  |
| 5 | Meng,Z.Q. | 2010 | Abdominal surgery for cancer | CHINA | Acupuncture | Control | 44 | 41 | 54.3 | 53.1 | A, B |
| 6 | Shang,H. | 2010 | Cesarean section | CHINA | Chewing Gum | Control | 193 | 195 | 19～44 | 19～44 | A, B |
| 7 | Chen Wei | 2011 | Abdominal operation | CHINA | Chewing Gum | Control | 70 | 70 | NR | NR | A, B, C |
| 8 | Han Zhi | 2011 | Myomectomy | CHINA | Chewing Gum | Control | 145 | 146 | NR | NR | A, B, C |
| 9 | Wang Shaoyuan | 2011 | Colectomy | CHINA | Chewing Gum | Control | 77 | 78 | 55.63 | 52.59 | A, B, C |
| 10 | Qiu Jinlin | 2011 | Abdominal operation | CHINA | Traditional herbal remedies +Acupuncture | Control | 100 | 100 | 46.5 | 47.2 | A, B |
|  |  |  |  |  | Traditional herbal remedies |  | 100 |  | 46.8 |  |  |
| 11 | Marwah, S. | 2012 | After ileostomy for perforation of typhoid ileum | India | Chewing Gum | Control | 50 | 50 | NR | NR | A, B |
| 12 | Müller, S. A. | 2012 | Colectomy | Germany | Coffee | Water | 40 | 40 | NR | NR | A, B, D |
| 13 | Chen Xiang | 2012 | Gastrectomy | CHINA | Chewing Gum | Control | 40 | 40 | 36-64 | 36-64 | A, B, C |
| 14 | Deng, G | 2013 | Colectomy | usa | Acupuncture | Control | 50 | 50 | 28-72 | 28-72 | A, B |
| 15 | Ertas, I. E | 2013 | Complete staging surgery for gynecological malignant tumor | Türkiye | Chewing Gum | Control | 46 | 47 | 56.2±10.9 | 55.7±11.2 | A, B, D |
| 16 | Jakkaew, B. | 2013 | Cesarean section | Thailand | Chewing Gum | Control | 55 | 55 | 67.4±9.7 | 68.5±10.6 | A, D |
| 17 | Ng, S. S. | 2013 | Colorectal surgery | Hong Kong | Electroacupuncture | Control | 55 | 55 | NR | NR | A, B, D |
|  |  |  |  |  | Acupuncture |  | 55 |  | NR |  |  |
| 18 | Ng,S.S.M. | 2013 | Laparoscopic surgery for colorectal cancer | HK | Electroacupuncture | Control | 55 | 55 | 67.4±9.7 | 68.5±10.6 | A, B, D |
| 19 | Sun Zhiyuan | 2013 | Laparotomy | CHINA | Chewing Gum | Control | 75 | 74 | 55.4±10.1 | 52.7±11.2 | A, B, C |
| 20 | Wanxidi | 2013 | Open cervical cancer root Treat surgery | CHINA | Chewing Gum | Control | 50 | 50 | 49.4±10.3 | 49.4±10.3 | A, B, C |
| 21 | Boelens, P. G. | 2014 | Major Rectal Surgery | Netherlands | Early Enteral Nutrition | Early Parenteral Nutrition | 61 | 62 | 64.0±1.4 | 65.0±1.2 | C |
| 22 | Shuhua Han | 2014 | Cesarean section | CHINA | Traditional herbal remedies | Control | 40 | 43 | 26.4±3.8 | 28.4±4.3 | A, B |
|  |  |  |  |  | Traditional herbal remedies +Acupuncture |  | 49 | 43 | 25.7±4.5 |  |  |
| 23 | Ziqiang Huang | 2014 | Abdominal operation | CHINA | Acupuncture | Traditional herbal remedies | 30 | 30 | 49.8±5.62 | 45.3±4.75 | A |
| 24 | OV Ajuzieogu | 2014 | Cesarean section | Nigeria | Chewing Gum | Control | 90 | 90 | 25.0±6.4 | 25.5±6.0 | A, B, C |
| 25 | Amelia M. Jernigan | 2014 | Laparotomy for benign gynecologic surgery | USA | Chewing Gum | Control | 51 | 58 | 42.8±8.7 | 42.1±10.6 | A, B, D |
| 26 | Wei Li | 2015 | Gynaecological surgery | CHINA | Chewing Gum | Control | 72 | 72 | 41.9±10 | 44.9±9.1 | A, B, C |
| 27 | Guangkui H | 2016 | Post-operative staging of malignant tumours | CHINA | Chewing Gum | Control | 86 | 92 | 38.1±4.7 | 54.3±4.9 | A, B, C |
| 28 | Wei Li | 2016 | Laparotomy for gynecological malignancies surgery | CHINA | Chewing Gum | Control | 78 | 78 | 48±10 | 47±9 | A, B, C |
| 29 | Xingyu L | 2016 | Gynaecological surgery | CHINA | Chewing Gum | Control | 45 | 45 | 51.16±2.77 | 50.89±2.83 | A, B, C |
| 30 | Chunjuan S | 2016 | Cesarean section | CHINA | Traditional herbal remedies +Acupuncture | Control | 40 | 40 | 28.2±3.3 | 27.1±3.1 | A, B |
|  |  |  |  |  | Traditional herbal remedies | Control | 40 | 40 | 27.8±3.4 | 27.1±3.1 |  |
| 31 | Hannah Lee | 2016 | Liver transplantation | Korea | Chewing Gum | Control | 30 | 29 | 56.7±9.1 | 53.7±8.1 | A |
| 32 | Audrius Dulskas | 2015 | Elective Laparoscopic Left-Sided Colectomy | Lithuania | Coffee | Water | 32 | 32 | 67.3±6.8 | 66.3±9.1 | A, B |
|  |  |  |  |  | Coffee without caffeine |  | 32 | 32 | 62.4±10.8 |  |  |
| 33 | Yunhui Gong, MD | 2015 | Laparoscopic Surgery | CHINA | Chewing Gum | Control | 53 | 56 | 39.55±10.25 | 39.41±10.64 | A, B, C |
| 34 | Xue-Mei You | 2015 | Hepatectomy | CHINA | Chewing Gum | Control | 53 | 54 | 53(29–75) | 51(28–69) | A, B, C |
|  |  |  |  |  | Traditional herbal remedies +Acupuncture |  | 55 |  | 48(28–71) |  |  |
| 35 | C. Atkinson | 2016 | Colorectal resection | UK | Chewing Gum | Control | 199 | 185 | 65.5±14.1 | 66.9±11.6 | A, B, C |
| 36 | N. F. Shum, H. K. Choi | 2016 | Laparoscopic colorectal resection | CHINA | Chewing Gum | Control | 41 | 41 | 68±10 | 69±12 | A, B |
| 37 | Sacide Yildizeli Topcu | 2016 | Colorectal Surgery | Turkey | Chewing Gum | Control | 30 | 30 | >18 | >18 | A, B |
| 38 | Omar Vergara-Fernandez | 2016 | Colorectal Surgery | Mexico | Chewing Gum | Control | 32 | 32 | >18 | >18 | D |
| 39 | Faezeh Abadi | 2017 | Cesarean Section | Iran | Acupuncture | Control | 60 | 60 | 28.85±4.3 | 28.2±4.8 | A, B, C |
| 40 | Kemal Güngördük | 2017 | Surgery of gynecological cancer | Turkey | Coffee | Control | 58 | 56 | 56.6±10.1 | 53.1±11.4 | A, B, C, D |
| 41 | Yang, Y. | 2017 | After elective open proctectomy | China | Traditional herbal remedies+Acupuncture | Chewing Gum | 186 | 190 | 53.7±15.1 | 53.3±14.9 | B, C |
|  |  |  |  |  | Control |  | 189 |  | 54.1±16.2 |  |  |
| 42 | Suhua，Lang | 2017 | Cesarean section | China | Chewing Gum | Control | 60 | 60 | 26.1±2.32 | 25.6±2.77 | A, B |
| 43 | Ahmed, M. R. | 2018 | Cesarean section | Egypt | Chewing Gum | Early Enteral Nutrition | 100 | 100 | 26.2±6 | 25.4±2 | A, B, C, D |
|  |  |  |  |  | Control |  | 100 |  | 26.6±3 |  |  |
| 44 | De Leede, E. M. | 2018 | Abdominal operation | Netherlands | Chewing Gum | Control | 975 | 996 | 66(18–94) | 66(19–98) | A, B |
| 45 | Esfehani, R. J. | 2018 | Cesarean section | Iran | Chewing Gum | Control | 35 | 58 | 24.87±4.54 | 24.81±4.74 | A, B, C |
| 46 | Pattamatta, M. | 2018 | Colorectal surgery | Netherlands | Chewing Gum | Control | 58 | 62 | 66±9 | 67±11 | D |
| 47 | Rabiepoor, S. | 2018 | Cesarean section | Iran | Coffee | Control | 50 | 50 | 28.46±5.35 | 28.22±5.14 | A, B, C |
| 48 | Yang, P. | 2018 | Elective open proctectomy | China | Chewing Gum | Control | 43 | 46 | 62.70±7.84 | 63.37±7.23 | A, B, C |
| 49 | Duzhang | 2018 | Colorectal surgery | China | Chewing Gum | Control | 63 | 57 | 46.53±6.42 | 48.74±6.45 | A, B |
| 50 | Hasler-Gehrer, S. | 2019 | Elective colorectal surgery | Switzerland | Coffee | Tea | 56 | 59 | 63(56–73) | 63(56–73) | B, C |
| 51 | Linhuis | 2019 | Gynecological benign tumor surgery | China | Electroacupuncture | Control | 30 | 30 | 34.57±8.29 | 35.50±8.80 | A, B |
|  |  |  |  |  | Accelerated rehabilitation |  | 30 |  | 35.5±8.0 |  |  |
| 52 | Wei Qiaoling | 2019 | Aparoscopic radical resection of colorectal cancer | China | Electroacupuncture | Control | 52 | 52 | 60.21±9.74 | 60.22±9.83 | A, B, D |
|  |  |  |  |  | Electroacupuncture +Chewing Gum |  | 52 |  | 60.21±9.78 |  |  |
| 53 | Bozkurt Koseoglu, S. | 2020 | Cesarean section | Türkiye | Coffee | Control | 51 | 52 | 28.70±5.42 | 29.25±5.74 | A, B, C, D |
| 54 | Gungorduk, K. | 2020 | Hysterectomy and bilateral salpingectomy were performed | Türkiye | Coffee | Water | 49 | 47 | 59(46–75) | 61(45–75) | A, B, C, D |
| 55 | Yuan, H. C. | 2020 | Laparoscopic Common Bile Duct Exploration | China | Control | Acupuncture+ Early Enteral Nutrition | 50 | 50 | 56.74±7.45 | 57.30±6.84 | A |
|  |  |  |  |  | Early Enteral Nutrition | Acupuncture | 50 | 50 | 57.14±8.31 | 59.94±7.72 |  |
| 56 | Wang Mei | 2020 | Abdominal operation | China | Chewing Gum | Control | 41 | 41 | 52.00±13.43 | 50.34±12.98 | A, B, C |
| 57 | Bhatti, S. | 2021 | Reversal of stoma | Pakistan | Chewing Gum | Control | 50 | 50 | 26.12(±7.1) | 28.80(±10.5) | A, B, C |
| 58 | Gao, W. | 2021 | Apen colorectal surgery | China | Electroacupuncture | Acupuncture | 303 | 307 | 62.0(53.0-69.0) | 63.0(54.0-70.0) | B, C |
| 59 | Zhang Jinping | 2021 | Laparoscopic radical resection of colorectal cancer | China | Water | Coffee | 94 | 94 | 55．9±13．6 | 56．1±11．6 | A |
| 60 | Hsu, Y. C. | 2022 | Apen colorectal surgery | China | Chewing Gum | Control | 30 | 30 | 59.57±9.56 | 58.07±9.47 | B |
| 61 | Sophie Hogan | 2022 | Pelvic exenteration surgery | Australia | Early Enteral Nutrition | Early Parenteral Nutrition | 35 | 40 | 61±11 | 63±13 | A |
| 62 | Yusuke | 2015 | Total Gastrectomy | Japan | Traditional herbal remedies | Control | 41 | 40 | 63.4±8.9 | 63.7±9.2 | B |
| 63 | Marc | 2006 | Major Colorectal Surgery | France | Intrathecal analgesia | Control | 26 | 26 | 78±5 | 77±5 |  |
| 64 | Varisara | 2006 | Cesarean Section | Thailand | Early Enteral Nutrition | Control | 107 | 93 | 29.6+5.7 | 29.2+6.1 | B |
| 65 | K. Charoenkwan | 2005 | Cesarean Section | Thailand | Early Enteral Nutrition | Control | 91 | 78 | NR | NR | A, D |
| 66 | John | 1991 | Colorectal Surgery | USA | Antemetic | Control | 40 | 53 | 59.5 | 59.5 | D |
| 67 | Chen | 2005 | colorectal surgery | China | Intravenous analgesia | Intravenous analgesia +Non-steroidal anti-inflammatory agents | 35 | 39 | 68 | 64.5 | B |
| 68 | Gong | 2016 | gastrointestinal surgery | China | Gastric prokinetic agents（High-dose） | Control | 45 | 42 | 37 | 38 | A, B, D |
| 69 | Shigeoki | 2008 | Postoperative gastric cancer | Japan | Seprafilm Adhesion Barrier | Control | 70 | 74 | 64 | 68 | A, B, D |
| 70 | Jin | 2012 | open gastrectomy for stomach cancer | Korea | Intravenous analgesia | Control | 24 | 23 | 55.2±8 | 56.4±7 | A, B |
| 71 | Sung | 2019 | radical gastrectomy | Korea | Antiadhesive agent | Control | 109 | 105 | 62.2±11.0 | 58.2±11.2 | A |
| 72 | takaaki | 2015 | Open Colectomy for Left-Sided Colorectal Cancer | Japan | Chewing Gum | Control | 21 | 22 | 66.4±13.9 | 68.0±9.6 | A, B |
| 73 | Isabelle | 2002 | Colectomy | France | Mechanical Massage of the Abdominal Wall | Control | 25 | 25 | 52±5 | 60±6 | A |
| 74 | Sung-Min | 2013 | laparoscopic rectal surgery | Korea | Accelerated rehabilitation | Control | 52 | 46 | 61.2±10.8 | 61.7±10.8 | A, B, D |
| 75 | Gabriela | 2016 | appendectomy | Canada | Chewing Gum | Control | 21 | 20 | 10.0(7.5-12) | 9.5(6-11.5) | A, B, D |
| 76 | Yao Lu | 2021 | abdominal surgery | China | Highly selective α2-adrenergic receptor agonist | Control | 344 | 331 | 70.1±5.8 | 70.4±6.5 | A, B, D |
| 77 | Susan | 2000 | Gynecologic Surgery | USA | Early Enteral Nutrition | Control | 67 | 72 | NR | NR | A. B |
| 78 | Okada | 2016 | pancreaticoduodenectomy | Japan | Traditional herbal remedies | Control | 104 | 103 | 68.9±8.4 | 64.9±11.3 | A, D |
| 79 | Michele | 2016 | laparoscopic cholecystectomy | Brazil | Infusion oflocal anesthetic | Control | 21 | 22 | 43.77±12.55 | 46.09±11.50 | A |
| 80 | Dolar | 2001 | Cesarean | USA | Early enteral nutrition | Control | 60 | 60 | 26.5±6.2 | 27.0±5.9 | B |
| 81 | Jingjing Qiu | 2022 | hepatectomy | China | Accelerated rehabilitation + Early enteral nutrition | Control | 54 | 54 | 49.12±9.33 | 49.19±9.24 | A, B, C |
| 82 | Ángela | 2021 | Ileostomy Closure | Spain | Stimulation with Probiotics before Surgery | Control | 34 | 35 | 65(45–81) | 68(41–80) | D |
| 83 | Irinel | 2010 | Partial Colectomy | Romania | Ghrelin receptor agonist （High-dose） | Control | 25 | 68 | 60±14 | 55±16 | A, D |
|  |  |  |  |  | Ghrelin receptor agonist （Low-dose） |  | 25 |  | 61±14 |  |  |
| 84 | Brian | 2010 | Open Colon Resection | USA | Infusion oflocal anesthetic | Epidural analgesia | 22 | 20 | 52(40-62) | 49(36-54) | A, B, D |
| 85 | Akiko | 2001 | abdominal surgery | USA | Opioid antagonist（High-dose） | Control | 26 | 26 | 56±9 | 54±12 | A, B, D |
|  |  |  |  |  | Opioid antagonist（Low-dose） |  | 26 |  | 49±13 |  |  |
| 86 | Teoh | 2007 | Caesarean delivery | Singapore | Early enteral nutrition | Control | 98 | 98 | 32.8±4.3 | 31.8±4.1 | A, B, D |
| 87 | Tollesson | 1991 | cholecystectomy | Sweden | Gastric prokinetic agents（High-dose） | Control | 20 | 20 | 55±3 | 45±3 | A, B |
| 88 | Heijkant | 2015 | colorectal surgery | Netherlands | Chewing Gum | Dermal patch | 58 | 62 | 66±9 | 67±11 | A |
| 89 | Kozo | 2015 | Total Gastrectomy | Japan | Traditional herbal remedies | Control | 96 | 99 | 68（33-83） | 67（28-84） | A, B |
| 90 | Zingg | 2008 | colorectal surgery | Switzerland | Laxative | Control | 83 | 86 | 67.9±13.2 | 66.4±14.5 | A, B, D |
| 91 | Jila | 2015 | cesarean | Iran | Antemetic | Control | 343 | 353 | 27.7±4.7 | 27.3±4.9 | A, B |
| 92 | Amir | 2018 | colon and rectal operations | USA | Intravenous analgesia | Control | 47 | 50 | 62.1±6.7 | 63.4±12.8 | A |
| 93 | Jin | 2015 | Laparoscopic Gastrectomy | Korea. | Highly selective α2-adrenergic receptor agonist | Control | 44 | 46 | 55.1±9.0 | 55.4±11.0 | A, D |
| 94 | Jin | 2017 | laparoscopic gastrectomy | Korea. | Epidural analgesia | Intravenous analgesia | 41 | 42 | 51.7±10.7 | 54.1±10.5 | B |
| 95 | Victor | 2006 | Intestinal Resection | USA | Seprafilm Adhesion Barrier | Control | 840 | 861 | 46.3±14.5 | 44.7±15.1 | A |
| 96 | Daniël | 2017 | colorectal surgery | Netherlands | Nicotine chewing gum | Chewing Gum | 20 | 20 | 69.00（62.50–70.00） | 67.50（60.75–74.75） | A, B |
| 97 | Woon | 2019 | colorectal surgery | Korea | Antiadhesive agent | Control | 155 | 166 | 60.3 | 62.5 | A |
|  |  |  |  |  | Seprafilm Adhesion Barrier |  | 167 |  | 63.2 |  |  |
| 98 | Evan | 2006 | open colectomy | USA | Chewing gum | Control | 22 | 21 | 62±14 | 58±15 | A. B |
|  |  |  |  |  | Accupressure bracelet worn |  | 23 |  | 54±11 |  |  |
| 99 | Chalaithorn | 2016 | cesarean delivery | Thailand | Early enteral nutrition | Control | 38 | 40 | 31.0(25.8–34.0) | 29.0(24.3–32.0) | A |
|  |  |  |  |  | Early enteral nutrition +Gastric prokinetic agents |  | 39 |  | 29.0(24.0–34.0) |  |  |
| 100 | Kazuhiro | 2008 | Hand-Assisted Laparoscopic Colectomy | Japan | Gastric prokinetic agents | Control | 20 | 20 | 64.2(35–82) | 70.6(55–85) | A, B |
| 101 | Ngowe | 2010 | Open Appendectomy | Cameroon | Chewing Gum | Control | 23 | 23 | 42.4±8.6 | 43.7±10 | A, B |
| 102 | Alessandra | 2021 | ovarian cancer cytoreductive surgery | Italy | Intestinal isolation bag | Control | 48 | 44 | 57.2±15.3 | 59.0±10.9 | A, B |
| 103 | Gota | 2019 | elective surgery for colon cancer | Japan | Seprafilm Adhesion Barrier | Control | 166 | 179 | 69 | 70 | A |
| 104 | Stewat | 1998 | ELECTIVE OPEN COLORECTAL RESECTIONS | Australia | Early enteral nutrition | Control | 40 | 40 | 58(25-89) | 59(17-88) | A, B, D |
| 105 | Zingg | 2009 | laparoscopic colorectal resection | Switzerland | Epidural analgesia | Control | 39 | 36 | 64.8±2.0 | 60.6±2.3 | A, B,D |

A: The incidence of postoperative ileus; B: The first exhaust time; C: The first time of bowel movement; D: The normal eating time

S2 Table of pairwise comparisons of interventions to prevent the incidence of postoperative ileus MD (95% CI UP, DOWN)

| IA | Coffee | EACU+CG | Tea | CG | GPA | NCG | AR+EEN | AA | SAB | THR | AR | ACU+EEN | EACU | EEN | SPBS | DP | Water | THR+ACU | Control | ACU | EPN |
| --- | --- | --- | --- | --- | --- | --- | --- | --- | --- | --- | --- | --- | --- | --- | --- | --- | --- | --- | --- | --- | --- |
| IA |  |  |  |  |  |  |  |  |  |  |  |  |  |  |  |  |  |  |  |  |  |
| -0.66  (-3.19,1.87) | Coffee |  |  |  |  |  |  |  |  |  |  |  |  |  |  |  |  |  |  |  |  |
| -1.08  (-4.30,2.13) | -0.42  (-3.03,2.18) | EACU+CG |  |  |  |  |  |  |  |  |  |  |  |  |  |  |  |  |  |  |  |
| -0.97  (-4.78,2.84) | -0.31  (-3.63,3.00) | 0.11  (-3.75,3.97) | Tea |  |  |  |  |  |  |  |  |  |  |  |  |  |  |  |  |  |  |
| -1.61  (-3.88,0.65) | -0.95  (-2.21,0.31) | -0.53  (-2.88,1.82) | -0.64  (-3.73,2.45) | CG |  |  |  |  |  |  |  |  |  |  |  |  |  |  |  |  |  |
| -1.62  (-4.18,0.94) | -0.96  (-2.70,0.77) | -0.54  (-3.17,2.10) | -0.65  (-3.98,2.69) | -0.01  (-1.33,1.31) | GPA |  |  |  |  |  |  |  |  |  |  |  |  |  |  |  |  |
| -1.61  (-4.36,1.14) | -0.95  (-2.96,1.05) | -0.53  (-3.35,2.29) | -0.64  (-4.10,2.82) | -0.00  (-1.56,1.56) | 0.01  (-2.03,2.05) | NCG |  |  |  |  |  |  |  |  |  |  |  |  |  |  |  |
| -1.45  (-5.44,2.55) | -0.79  (-4.31,2.74) | -0.36  (-4.41,3.68) | -0.47  (-5.01,4.06) | 0.17  (-3.17,3.50) | 0.17  (-3.37,3.72) | 0.17  (-3.52,3.85) | AR+EEN |  |  |  |  |  |  |  |  |  |  |  |  |  |  |
| -1.80  (-4.14,0.55) | -1.14  (-2.54,0.26) | -0.72  (-3.14,1.71) | -0.83  (-4.00,2.35) | -0.19  (-1.02,0.64) | -0.18  (-1.63,1.27) | -0.19  (-1.95,1.58) | -0.35  (-3.75,3.04) | AA |  |  |  |  |  |  |  |  |  |  |  |  |  |
| -2.22  (-4.51,0.08) | -1.56  (-2.86,-0.25) | -1.13  (-3.51,1.24) | -1.24  (-4.38,1.89) | -0.60  (-1.25,0.04) | -0.60  (-1.96,0.77) | -0.60  (-2.29,1.08) | -0.77  (-4.13,2.59) | -0.42  (-1.25,0.42) | SAB |  |  |  |  |  |  |  |  |  |  |  |  |
| -2.22  (-4.57,0.12) | -1.56  (-2.96,-0.17) | -1.14  (-3.57,1.29) | -1.25  (-4.42,1.92) | -0.61  (-1.43,0.20) | -0.60  (-2.06,0.85) | -0.61  (-2.37,1.15) | -0.78  (-4.17,2.62) | -0.42  (-1.45,0.60) | -0.01  (-0.90,0.88) | THR |  |  |  |  |  |  |  |  |  |  |  |
| -2.53  (-6.73,1.67) | -1.87  (-5.62,1.88) | -1.45  (-5.58,2.68) | -1.56  (-6.27,3.15) | -0.92  (-4.50,2.66) | -0.91  (-4.68,2.86) | -0.92  (-4.82,2.99) | -1.08  (-5.95,3.78) | -0.73  (-4.36,2.90) | -0.31  (-3.91,3.28) | -0.31  (-3.94,3.32) | AR |  |  |  |  |  |  |  |  |  |  |
| -2.62  (-6.62,1.38) | -1.96  (-5.49,1.57) | -1.54  (-5.51,2.44) | -1.65  (-6.19,2.89) | -1.01  (-4.36,2.34) | -1.00  (-4.56,2.55) | -1.01  (-4.70,2.68) | -1.17  (-5.87,3.52) | -0.82  (-4.22,2.58) | -0.40  (-3.77,2.96) | -0.40  (-3.80,3.01) | -0.09  (-4.89,4.71) | ACU+EEN |  |  |  |  |  |  |  |  |  |
| -2.50  (-5.13,0.13) | -1.84  (-3.68,0.00) | -1.41  (-3.73,0.90) | -1.52  (-4.92,1.87) | -0.89  (-2.34,0.57) | -0.88  (-2.76,1.00) | -0.89  (-3.02,1.25) | -1.05  (-4.65,2.55) | -0.70  (-2.27,0.88) | -0.28  (-1.77,1.21) | -0.27  (-1.85,1.30) | 0.03  (-3.52,3.59) | 0.12  (-3.31,3.55) | EACU |  |  |  |  |  |  |  |  |
| -2.45  (-4.74,-0.15) | -1.79  (-3.10,-0.48) | -1.36  (-3.74,1.01) | -1.47  (-4.61,1.66) | -0.83  (-1.51,-0.16) | -0.83  (-2.19,0.54) | -0.83  (-2.53,0.86) | -1.00  (-4.36,2.36) | -0.65  (-1.55,0.26) | -0.23  (-0.99,0.53) | -0.22  (-1.13,0.68) | 0.08  (-3.51,3.68) | 0.17  (-3.16,3.51) | 0.05  (-1.43,1.53) | EEN |  |  |  |  |  |  |  |
| -2.47  (-5.04,0.11) | -1.81  (-3.56,-0.05) | -1.38  (-4.04,1.27) | -1.50  (-4.84,1.85) | -0.86  (-2.20,0.49) | -0.85  (-2.65,0.95) | -0.86  (-2.92,1.20) | -1.02  (-4.58,2.53) | -0.67  (-2.14,0.81) | -0.25  (-1.64,1.14) | -0.24  (-1.72,1.23) | 0.06  (-3.72,3.84) | 0.15  (-3.41,3.72) | 0.03  (-1.87,1.93) | -0.02  (-1.42,1.37) | SPBS |  |  |  |  |  |  |
| -2.54  (-5.07,-0.02) | -1.88  (-3.56,-0.20) | -1.46  (-4.06,1.14) | -1.57  (-4.85,1.71) | -0.93  (-2.04,0.18) | -0.92  (-2.65,0.80) | -0.93  (-2.84,0.98) | -1.10  (-4.62,2.42) | -0.74  (-2.13,0.64) | -0.33  (-1.61,0.96) | -0.32  (-1.70,1.06) | -0.01  (-3.76,3.73) | 0.08  (-3.45,3.60) | -0.05  (-1.87,1.78) | -0.10  (-1.40,1.20) | -0.08  (-1.82,1.67) | DP |  |  |  |  |  |
| -2.79  (-6.19,0.61) | -2.13  (-4.40,0.14) | -1.70  (-5.16,1.76) | -1.82  (-5.83,2.20) | -1.18  (-3.77,1.42) | -1.17  (-4.03,1.69) | -1.18  (-4.21,1.85) | -1.34  (-5.53,2.85) | -0.99  (-3.66,1.68) | -0.57  (-3.19,2.05) | -0.56  (-3.23,2.10) | -0.26  (-4.64,4.13) | -0.17  (-4.37,4.03) | -0.29  (-3.21,2.63) | -0.34  (-2.97,2.28) | -0.32  (-3.19,2.55) | -0.24  (-3.07,2.58) | Water |  |  |  |  |
| -2.64  (-5.20,-0.08) | -1.98  (-3.72,-0.24) | -1.56  (-4.20,1.08) | -1.67  (-5.00,1.67) | -1.03  (-2.34,0.28) | -1.02  (-2.80,0.76) | -1.03  (-3.07,1.01) | -1.19  (-4.74,2.35) | -0.84  (-2.30,0.62) | -0.42  (-1.79,0.94) | -0.42  (-1.66,0.82) | -0.11  (-3.89,3.66) | -0.02  (-3.58,3.54) | -0.14  (-2.03,1.74) | -0.19  (-1.57,1.18) | -0.17  (-1.98,1.63) | -0.10  (-1.81,1.62) | 0.15  (-2.72,3.01) | THR+ACU |  |  |  |
| -2.56  (-4.79,-0.33) | -1.90  (-3.10,-0.71) | -1.48  (-3.80,0.84) | -1.59  (-4.68,1.50) | -0.95  (-1.35,-0.55) | -0.94  (-2.20,0.31) | -0.95  (-2.56,0.66) | -1.12  (-4.43,2.20) | -0.76  (-1.49,-0.04) | -0.35  (-0.88,0.18) | -0.34  (-1.07,0.39) | -0.03  (-3.59,3.52) | 0.06  (-3.27,3.38) | -0.07  (-1.46,1.33) | -0.12  (-0.66,0.42) | -0.10  (-1.38,1.19) | -0.02  (-1.20,1.16) | 0.22  (-2.34,2.79) | 0.08  (-1.19,1.34) | Control |  |  |
| -2.85  (-5.57,-0.14) | -2.19  (-4.15,-0.23) | -1.77  (-4.21,0.67) | -1.88  (-5.34,1.58) | -1.24  (-2.84,0.36) | -1.23  (-3.23,0.77) | -1.24  (-3.48,0.99) | -1.41  (-5.07,2.25) | -1.05  (-2.77,0.66) | -0.64  (-2.28,1.00) | -0.63  (-2.34,1.08) | -0.32  (-3.96,3.31) | -0.23  (-3.67,3.21) | -0.36  (-1.18,0.47) | -0.41  (-2.03,1.22) | -0.38  (-2.40,1.63) | -0.31  (-2.26,1.64) | -0.07  (-3.07,2.94) | -0.21  (-2.21,1.79) | -0.29  (-1.84,1.26) | ACU |  |
| -3.02  (-5.45,-0.60) | -2.36  (-3.89,-0.84) | -1.94  (-4.44,0.56) | -2.05  (-5.28,1.18) | -1.41  (-2.45,-0.38) | -1.40  (-2.98,0.17) | -1.41  (-3.28,0.46) | -1.58  (-5.02,1.87) | -1.22  (-2.42,-0.03) | -0.81  (-1.90,0.28) | -0.80  (-2.00,0.40) | -0.49  (-4.17,3.19) | -0.40  (-3.83,3.02) | -0.53  (-2.20,1.15) | -0.58  (-1.36,0.21) | -0.56  (-2.15,1.04) | -0.48  (-2.00,1.04) | -0.24  (-2.97,2.50) | -0.38  (-1.97,1.20) | -0.46  (-1.41,0.49) | -0.17  (-1.98,1.63) | EPN |

S3 Table of pairwise comparisons of the first exhaust time (MD, 95% CI)

| MMAW | ACU+EEN | EA | IIB | OA-H | THR+ACU | Tea | GPA-L | IA | CG | ACU | NCG | LAX | OA-L | EACU | SPBS | Water | AR | GPA-H | ACU | AR+EEN | Coffee | SAB | EEN+GPA | THR | ABW | Control | EACU+CG | AA |
| --- | --- | --- | --- | --- | --- | --- | --- | --- | --- | --- | --- | --- | --- | --- | --- | --- | --- | --- | --- | --- | --- | --- | --- | --- | --- | --- | --- | --- |
| MMAW |  |  |  |  |  |  |  |  |  |  |  |  |  |  |  |  |  |  |  |  |  |  |  |  |  |  |  |  |
| -16.38  (-39.69,6.94) | ACU+EEN |  |  |  |  |  |  |  |  |  |  |  |  |  |  |  |  |  |  |  |  |  |  |  |  |  |  |  |
| -21.82  (-47.93,4.30) | -5.44  (-28.35,17.47) | EA |  |  |  |  |  |  |  |  |  |  |  |  |  |  |  |  |  |  |  |  |  |  |  |  |  |  |
| -21.84  (-48.40,4.73) | -5.4  6 (-28.89,17.97) | -0.02  (-26.24,26.20) | IIB |  |  |  |  |  |  |  |  |  |  |  |  |  |  |  |  |  |  |  |  |  |  |  |  |  |
| -22.20  (-50.18,5.79) | -5.82  (-30.84,19.20) | -0.38  (-28.03,27.27) | -0.36  (-28.44,27.72) | OA-H |  |  |  |  |  |  |  |  |  |  |  |  |  |  |  |  |  |  |  |  |  |  |  |  |
| -28.49  (-48.75,-8.23) | -12.11  (-27.97,3.75) | -6.67  (-26.47,13.13) | -6.65  (-27.04,13.74) | -6.29  (-28.49,15.91) | THR+ACU |  |  |  |  |  |  |  |  |  |  |  |  |  |  |  |  |  |  |  |  |  |  |  |
| -28.98  (-51.07,-6.88) | -12.60  (-30.79,5.60) | -7.16  (-28.83,14.51) | -7.14  (-29.35,15.08) | -6.78  (-30.67,17.11) | -0.49  (-14.52,13.54) | Tea |  |  |  |  |  |  |  |  |  |  |  |  |  |  |  |  |  |  |  |  |  |  |
| -27.29  (-56.75,2.17) | -10.92  (-37.58,15.74) | -5.48  (-34.62,23.67) | -5.46  (-35.01,24.09) | -5.10  (-35.93,25.73) | 1.19  (-22.85,25.23) | 1.68  (-23.92,27.29) | GPA-L |  |  |  |  |  |  |  |  |  |  |  |  |  |  |  |  |  |  |  |  |  |
| -30.94  (-51.97,-9.91) | -14.56  (-31.45,2.32) | -9.12  (-25.30,7.05) | -9.10  (-30.25,12.05) | -8.74  (-31.65,14.16) | -2.45  (-14.78,9.88) | -1.96 (-17.12,13.20) | -3.64  (-28.33,21.04) | IA |  |  |  |  |  |  |  |  |  |  |  |  |  |  |  |  |  |  |  |  |
| -32.91  (-51.84,-13.98) | -16.54  (-30.70,-2.37) | -11.10  (-29.53,7.34) | -11.08  (-30.14,7.99) | -10.72  (-31.71,10.28) | -4.43  (-12.44,3.59) | -3.94  (-15.80,7.93) | -5.62  (-28.55,17.31) | -1.97  (-11.97,8.02) | CG |  |  |  |  |  |  |  |  |  |  |  |  |  |  |  |  |  |  |  |
| -33.29  (-53.20,-13.37) | -16.91  (-31.24,-2.58) | -11.47  (-30.92,7.98) | -11.45  (-31.50,8.60) | -11.09  (-32.98,10.80) | -4.80  (-14.86,5.27) | -4.31  (-17.89,9.27) | -5.99  (-29.74,17.76) | -2.35  (-14.10,9.40) | -0.37  (-7.73,6.98) | ACU |  |  |  |  |  |  |  |  |  |  |  |  |  |  |  |  |  |  |
| -32.91  (-59.83,-5.99) | -16.54  (-40.35,7.28) | -11.10  (-37.67,15.48) | -11.08  (-38.09,15.94) | -10.72  (-39.13,17.69) | -4.43  (-25.18,16.33) | -3.94  (-26.46,18.58) | -5.62  (-35.48,24.25) | -1.97  (-23.57,19.62) | 0.00  (-19.14,19.14) | 0.37  (-20.13,20.88) | NCG |  |  |  |  |  |  |  |  |  |  |  |  |  |  |  |  |  |
| -33.60  (-60.12,-7.07) | -17.22  (-40.60,6.16) | -11.78  (-37.96,14.39) | -11.76  (-38.39,14.87) | -11.40  (-39.44,16.64) | -5.11  (-25.45,15.23) | -4.62  (-26.79,17.54) | -6.30  (-35.81,23.21) | -2.66  (-23.76,18.44) | -0.68  (-19.69,18.33) | -0.31  (-20.31,19.68) | -0.68  (-27.66,26.29) | LAX |  |  |  |  |  |  |  |  |  |  |  |  |  |  |  |  |
| -34.20  (-62.58,-5.82) | -17.82  (-43.29,7.64) | -12.38  (-40.43,15.67) | -12.36  (-40.83,16.11) | -12.00  (-30.92,6.92) | -5.71  (-28.41,16.99) | -5.22  (-29.58,19.13) | -6.90  (-38.09,24.29) | -3.26  (-26.65,20.13) | -1.28  (-22.81,20.24) | -0.91  (-23.31,21.49) | -1.28  (-30.09,27.52) | -0.60  (-29.03,27.83) | OA-L |  |  |  |  |  |  |  |  |  |  |  |  |  |  |  |
| -34.52  (-55.07,-13.98) | -18.15  (-34.07,-2.22) | -12.71  (-32.80,7.38) | -12.69  (-33.36,7.99) | -12.33  (-34.79,10.14) | -6.04  (-17.47,5.40) | -5.55  (-20.03,8.93) | -7.23  (-31.51,17.05) | -3.58  (-16.38,9.21) | -1.61  (-10.53,7.31) | -1.24  (-11.03,8.55) | -1.61  (-22.73,19.51) | -0.93  (-21.55,19.69) | -0.33  (-23.28,22.63) | EACU |  |  |  |  |  |  |  |  |  |  |  |  |  |  |
| -43.20  (-343.37,256.98) | -26.82  (-326.73,273.09) | -21.38  (-321.53,278.76) | -21.36  (-321.54,278.82) | -21.00  (-321.31,279.31) | -14.71  (-314.40,284.98) | -14.22  (-314.04,285.60) | -15.90  (-316.36,284.55) | -12.26  (-312.00,287.49) | -10.28  (-309.89,289.32) | -9.9  1 (-309.58,289.76) | -10.28  (-310.50,289.93) | -9.60  (-309.78,290.58) | -9.00  (-309.35,291.35) | -8.67  (-308.38,291.04) | SPBS |  |  |  |  |  |  |  |  |  |  |  |  |  |
| -34.83  (-58.87,-10.79) | -18.46  (-38.97,2.06) | -13.02  (-36.67,10.63) | -12.99  (-37.14,11.15) | -12.63  (-38.33,13.06) | -6.34  (-23.30,10.61) | -5.86  (-23.23,11.52) | -7.54  (-34.83,19.76) | -3.89  (-21.76,13.98) | -1.92  (-17.23,13.39) | -1.55  (-18.10,15.01) | -1.92  (-26.43,22.59) | -1.23  (-25.34,22.87) | -0.63  (-26.76,25.50) | -0.31  (-17.61,17.00) | 8.37  (-291.61,308.34) | Water |  |  |  |  |  |  |  |  |  |  |  |  |
| -34.97  (-57.13,-12.80) | -18.59  (-36.77,-0.41) | -13.15  (-34.90,8.60) | -13.13  (-35.41,9.15) | -12.77  (-36.72,11.18) | -6.48  (-20.65,7.69) | -5.99  (-22.69,10.71) | -7.67  (-33.34,17.99) | -4.03  (-19.29,11.24) | -2.05  (-14.26,10.15) | -1.68  (-15.11,11.75) | -2.05  (-24.75,20.65) | -1.37  (-23.60,20.86) | -0.77  (-25.18,23.65) | -0.44  (-13.37,12.48) | 8.23  (-291.60,308.06) | -0.13  (-19.33,19.07) | AR |  |  |  |  |  |  |  |  |  |  |  |
| -35.16  (-55.35,-14.96) | -18.78  (-34.62,-2.94) | -13.34  (-33.08,6.40) | -13.32  (-33.65,7.01) | -12.96  (-35.11,9.19) | -6.67  (-17.53,4.19) | -6.18  (-20.17,7.81) | -7.86  (-31.09,15.37) | -4.22  (-16.45,8.02) | -2.24  (-10.35,5.86) | -1.87  (-12.08,8.34) | -2.24  (-23.03,18.54) | -1.56  (-21.83,18.72) | -0.96  (-23.61,21.69) | -0.63  (-12.01,10.75) | 8.04  (-291.65,307.73) | -0.32  (-17.22,16.57) | -0.19 (-14.28,13.90) | GPA-H |  |  |  |  |  |  |  |  |  |  |
| -35.29  (-54.96,-15.62) | -18.92  (-33.13,-4.70) | -13.48  (-32.67,5.72) | -13.45  (-33.26,6.35) | -13.09  (-34.76,8.57) | -6.80  (-16.60,2.99) | -6.32  (-19.51,6.88) | -8.00  (-31.54,15.54) | -4.35  (-15.68,6.98) | -2.38  (-8.94,4.19) | -2.01  (-10.57,6.56) | -2.38  (-22.61,17.86) | -1.69  (-21.44,18.05) | -1.09  (-23.27,21.08) | -0.77  (-10.59,9.06) | 7.91  (-291.75,307.56) | -0.46  (-16.71,15.79) | -0.33  (-13.51,12.86) | -0.14  (-9.84,9.57) | ACU |  |  |  |  |  |  |  |  |  |
| -37.44  (-62.36,-12.52) | -21.07  (-42.61,0.47) | -15.63  (-40.17,8.92) | -15.61  (-40.63,9.42) | -15.25  (-41.77,11.28) | -8.96  (-27.15,9.24) | -8.47  (-28.68,11.75) | -10.15  (-38.23,17.93) | -6.50  (-25.55,12.54) | -4.53  (-21.22,12.16) | -4.16  (-21.97,13.65) | -4.53  (-29.93,20.87) | -3.85  (-28.83,21.14) | -3.25  (-30.19,23.70) | -2.92  (-21.43,15.59) | 5.75  (-294.29,305.80) | -2.61  (-24.94,19.71) | -2.48  (-22.77,17.81) | -2.29  (-20.41,15.84) | -2.15  (-19.68,15.38) | AR+EEN |  |  |  |  |  |  |  |  |
| -36.92  (-57.62,-16.22) | -20.54  (-37.02,-4.07) | -15.10  (-35.35,5.15) | -15.08  (-35.91,5.75) | -14.72  (-37.33,7.88) | -8.43  (-20.19,3.32) | -7.94  (-20.29,4.40) | -9.62  (-34.03,14.79) | -5.98  (-19.02,7.06) | -4.01  (-13.23,5.21) | -3.63  (-14.80,7.53) | -4.01  (-25.25,17.24) | -3.32  (-24.10,17.45) | -2.72  (-25.82,20.37) | -2.40  (-14.64,9.85) | 6.28  (-293.44,306.00) | -2.09  (-14.31,10.14) | -1.95  (-16.76,12.85) | -1.76  (-13.42,9.90) | -1.63  (-12.33,9.08) | 0.52  (-18.16,19.21) | Coffee |  |  |  |  |  |  |  |
| -43.20  (-95.11,8.72) | -26.82  (-77.20,23.56) | -21.38  (-73.12,30.36) | -21.36  (-73.33,30.61) | -21.00  (-73.71,31.71) | -14.71  (-63.75,34.34) | -14.22  (-64.05,35.61) | -15.90  (-69.41,37.60) | -12.26  (-61.62,37.11) | -10.28  (-58.79,38.23) | -9.91  (-58.82,38.99) | -10.28  (-62.43,41.87) | -9.60  (-61.55,42.35) | -9.00  (-61.92,43.92) | -8.67  (-57.84,40.49) | -0.00  (-303.48,303.48) | -8.37  (-59.09,42.36) | -8.23  (-58.09,41.63) | -8.04  (-57.06,40.98) | -7.91  (-56.71,40.90) | -5.75  (-56.90,45.39) | -6.28  (-55.51,42.95) | SAB |  |  |  |  |  |  |
| -40.40  (-64.98,-15.81) | -24.02  (-44.84,-3.20) | -18.58  (-42.78,5.62) | -18.56  (-43.25,6.13) | -18.20  (-44.40,8.01) | -11.91  (-29.62,5.81) | -11.42  (-31.21,8.37) | -13.10  (-40.88,14.68) | -9.46  (-28.05,9.14) | -7.48  (-23.65,8.68) | -7.11  (-24.30,10.08) | -7.48  (-32.54,17.57) | -6.80  (-31.44,17.84) | -6.20  (-32.83,20.43) | -5.87  (-23.76,12.01) | 2.80  (-297.21,302.81) | -5.57  (-27.51,16.38) | -5.43  (-25.26,14.40) | -5.24  (-22.90,12.41) | -5.11  (-21.04,10.83) | -2.95  (-25.86,19.95) | -3.48 (-21.70,14.75) | 2.80  (-48.18,53.78) | EEN+GPA |  |  |  |  |  |
| -39.96  (-60.26,-19.67) | -23.59  (-39.32,-7.86) | -18.15  (-37.98,1.68) | -18.13  (-38.55,2.30) | -17.77  (-40.00,4.46) | -11.48  (-20.88,-2.08) | -10.99  (-25.10,3.12) | -12.67  (-36.73,11.39) | -9.02  (-21.40,3.35) | -7.05  (-15.32,1.22) | -6.68  (-16.07,2.71) | -7.05  (-27.90,13.80) | -6.37  (-26.73,14.00) | -5.77  (-28.50,16.96) | -5.44  (-16.78,5.90) | 3.23  (-296.46,302.93) | -5.13  (-22.13,11.87) | -5.00  (-19.18,9.18) | -4.81  (-15.73,6.11) | -4.67  (-14.47,5.12) | -2.52  (-20.75,15.71) | -3.05  (-14.86,8.77) | 3.23  (-45.82,52.29) | 0.43  (-17.30,18.16) | THR |  |  |  |  |
| -46.44  (-75.18,-17.70) | -30.06  (-55.92,-4.21) | -24.62  (-53.04,3.79) | -24.60  (-53.43,4.23) | -24.24  (-54.38,5.90) | -17.95  (-41.05,5.15) | -17.46  (-42.18,7.25) | -19.15  (-50.66,12.37) | -15.50  (-39.32,8.32) | -13.53  (-35.32,8.27) | -13.15  (-36.00,9.69) | -13.53  (-42.53,15.48) | -12.84  (-41.63,15.95) | -12.24  (-42.75,18.26) | -11.92  (-35.31,11.48) | -3.24  (-303.63,297.14) | -11.61  (-38.11,14.90) | -11.47  (-36.30,13.35) | -11.28  (-34.38,11.81) | -11.15  (-33.76,11.46) | -9.00  (-36.31,18.32) | -9.52  (-33.04,14.00) | -3.24  (-56.35,49.87) | -6.04  (-33.05,20.96) | -6.48  (-29.64,16.68) | ABW |  |  |  |
| -43.20  (-61.91,-24.48) | -26.82  (-40.72,-12.92) | -21.38  (-39.60,-3.17) | -21.36  (-40.22,-2.50) | -21.00  (-41.80,-0.20) | -14.71  (-22.47,-6.95) | -14.22  (-25.97,-2.48) | -15.90  (-38.65,6.85) | -12.26  (-21.84,-2.68) | -10.28  (-13.12,-7.45) | -9.91  (-16.73,-3.10) | -10.28  (-29.63,9.07) | -9.60  (-28.40,9.20) | -9.00  (-30.33,12.33) | -8.67  (-17.15,-0.20) | -0.00  (-299.59,299.59) | -8.37  (-23.45,6.72) | -8.23  (-20.10,3.64) | -8.04  (-15.64,-0.44) | -7.91  (-13.95,-1.86) | -5.75  (-22.21,10.70) | -6.28  (-15.12,2.57) | -0.00  (-48.43,48.43) | -2.80  (-18.74,13.14) | -3.23  (-11.08,4.61) | 3.24  (-18.56,25.05) | Control |  |  |
| -47.22  (-71.47,-22.97) | -30.85  (-51.47,-10.22) | -25.41  (-49.27,-1.54) | -25.39  (-49.74,-1.03) | -25.03  (-50.92,0.87) | -18.74  (-35.98,-1.49) | -18.25  (-37.63,1.14) | -19.93  (-47.41,7.56) | -16.28  (-34.44,1.87) | -14.31  (-29.98,1.36) | -13.94  (-30.46,2.59) | -14.31  (-39.05,10.43) | -13.63  (-37.94,10.69) | -13.03  (-39.35,13.30) | -12.70  (-28.12,2.72) | -4.03  (-304.01,295.96) | -12.39  (-33.96,9.18) | -12.26  (-31.12,6.61) | -12.07  (-29.26,5.12) | -11.93  (-28.32,4.45) | -9.78  (-32.33,12.77) | -10.30  (-28.08,7.47) | -4.03  (-54.85,46.80) | -6.83  (-28.94,15.28) | -7.26  (-24.49,9.97) | -0.78  (-27.49,25.92) | 4.03  (-11.39,19.45) | EACU+CG |  |
| -52.80  (-78.80,-26.79) | -36.42  (-59.21,-13.63) | -30.98  (-56.63,-5.33) | -30.96  (-57.07,-4.85) | -30.60  (-58.15,-3.05) | -24.31  (-43.96,-4.65) | -23.82  (-45.36,-2.28) | -25.50  (-54.55,3.54) | -21.86  (-42.30,-1.42) | -19.88  (-38.16,-1.61) | -19.51  (-38.81,-0.21) | -19.88  (-46.35,6.58) | -19.20  (-45.26,6.87) | -18.60  (-46.55,9.35) | -18.27  (-38.22,1.67) | -9.60  (-309.73,290.54) | -17.97  (-41.49,5.56) | -17.83  (-39.44,3.78) | -17.64  (-37.23,1.95) | -17.51  (-36.55,1.54) | -15.35  (-39.78,9.08) | -15.88  (-35.98,4.23) | -9.60  (-61.28,42.08) | -12.40  (-36.48,11.68) | -12.83  (-32.52,6.85) | -6.36  (-34.67,21.96) | 9.60  (-8.46,27.66) | -5.57  (-29.32,18.17) | AA |

S4 Table of pairwise comparisons of the first bowel movement time (MD, 95% CI)

| OA-H | THR+ACU | EACU+CG | LAX | OA-L | CWC | EA | EACU | GPA | THR | ABW | IA | IA+NSAIA | CG | NCG | ARA | SPBS | AR | EEN | Coffee | AR+EEN | SAB | IIB | ACU | Tea | Water | Control | EPN |
| --- | --- | --- | --- | --- | --- | --- | --- | --- | --- | --- | --- | --- | --- | --- | --- | --- | --- | --- | --- | --- | --- | --- | --- | --- | --- | --- | --- |
| OA-H |  |  |  |  |  |  |  |  |  |  |  |  |  |  |  |  |  |  |  |  |  |  |  |  |  |  |  |
| -9.47  (-49.82,30.88) | THR+ACU |  |  |  |  |  |  |  |  |  |  |  |  |  |  |  |  |  |  |  |  |  |  |  |  |  |  |
| -9.07  (-53.82,35.67) | 0.39  (-25.81,26.59) | EACU+CG |  |  |  |  |  |  |  |  |  |  |  |  |  |  |  |  |  |  |  |  |  |  |  |  |  |
| -12.20  (-59.51,35.11) | -2.73  (-33.11,27.65) | -3.12  (-39.14,32.89) | LAX |  |  |  |  |  |  |  |  |  |  |  |  |  |  |  |  |  |  |  |  |  |  |  |  |
| -10.00  (-38.32,18.32) | -0.53  (-41.73,40.66) | -0.93  (-46.44,44.58) | 2.20  (-45.83,50.23) | OA-L |  |  |  |  |  |  |  |  |  |  |  |  |  |  |  |  |  |  |  |  |  |  |  |
| -13.35  (-60.97,34.27) | -3.88  (-34.74,26.97) | -4.28  (-40.70,32.15) | -1.15  (-40.68,38.37) | -3.35  (-51.69,44.98) | CWC |  |  |  |  |  |  |  |  |  |  |  |  |  |  |  |  |  |  |  |  |  |  |
| -16.99  (-62.15,28.16) | -7.53  (-34.42,19.37) | -7.92  (-41.05,25.21) | -4.80  (-41.31,31.72) | -6.99  (-52.90,38.91) | -3.64  (-40.56,33.27) | EA |  |  |  |  |  |  |  |  |  |  |  |  |  |  |  |  |  |  |  |  |  |
| -18.39  (-59.47,22.70) | -8.92  (-28.19,10.35) | -9.31  (-32.34,13.71) | -6.19  (-37.54,25.16) | -8.39  (-50.30,33.53) | -5.04  (-36.85,26.77) | -1.39  (-29.38,26.59) | EACU |  |  |  |  |  |  |  |  |  |  |  |  |  |  |  |  |  |  |  |  |
| -10.50  (-95.37,74.37) | -1.03  (-77.76,75.70) | -1.42  (-80.56,77.71) | 1.70  (-78.91,82.31) | -0.50  (-85.77,84.77) | 2.85  (-77.94,83.64) | 6.50  (-72.87,85.86) | 7.89  (-69.23,85.01) | GPA |  |  |  |  |  |  |  |  |  |  |  |  |  |  |  |  |  |  |  |
| -22.26  (-63.56,19.03) | -12.80  (-28.32,2.73) | -13.19  (-40.83,14.45) | -10.07  (-41.69,21.56) | -12.26  (-54.38,29.85) | -8.91  (-41.00,23.17) | -5.27  (-33.56,23.02) | -3.88  (-25.07,17.32) | -11.77  (-89.00,65.47) | THR |  |  |  |  |  |  |  |  |  |  |  |  |  |  |  |  |  |  |
| -22.80  (-69.88,24.29) | -13.33  (-43.30,16.64) | -13.72  (-49.45,22.00) | -10.60  (-49.48,28.28) | -12.80  (-60.61,35.01) | -9.45  (-48.71,29.81) | -5.80  (-42.03,30.42) | -4.41  (-35.42,26.60) | -12.30  (-92.78,68.18) | -0.53  (-31.79,30.73) | ABW |  |  |  |  |  |  |  |  |  |  |  |  |  |  |  |  |  |
| -23.65  (-64.64,17.33) | -14.19  (-33.34,4.96) | -14.58  (-41.78,12.62) | -11.45  (-42.67,19.77) | -13.65  (-55.47,28.16) | -10.30  (-42.00,21.39) | -6.66  (-34.50,21.18) | -5.27  (-25.90,15.37) | -13.15  (-90.22,63.91) | -1.39  (-22.43,19.65) | -0.86  (-31.73,30.02) | IA |  |  |  |  |  |  |  |  |  |  |  |  |  |  |  |  |
| -24.08  (-64.94,16.79) | -14.61  (-33.44,4.22) | -15.00  (-42.00,12.00) | -11.88  (-42.94,19.18) | -14.08  (-55.77,27.62) | -10.73  (-42.26,20.81) | -7.08  (-34.75,20.58) | -5.69  (-26.05,14.67) | -13.58  (-90.58,63.43) | -1.81  (-22.59,18.97) | -1.28  (-32.00,29.44) | -0.42  (-17.73,16.88) | IA+NSAIA |  |  |  |  |  |  |  |  |  |  |  |  |  |  |  |
| -25.00  (-63.60,13.60) | -15.54  (-28.47,-2.61) | -15.93  (-39.36,7.50) | -12.81  (-40.82,15.21) | -15.00  (-54.48,24.48) | -11.65  (-40.19,16.88) | -8.01  (-32.20,16.18) | -6.62  (-21.93,8.70) | -14.51  (-90.33,61.32) | -2.74  (-18.50,13.02) | -2.21  (-29.49,25.08) | -1.35  (-16.40,13.70) | -0.93  (-15.64,13.79) | CG |  |  |  |  |  |  |  |  |  |  |  |  |  |  |
| -25.00  (-79.76,29.75) | -15.54  (-56.47,25.39) | -15.93  (-61.28,29.42) | -12.81  (-60.69,35.08) | -15.00  (-70.38,40.37) | -11.65  (-59.84,36.53) | -8.01  (-53.76,37.74) | -6.62  (-48.36,35.12) | -14.51  (-99.70,70.68) | -2.74  (-44.65,39.17) | -2.21  (-49.67,45.25) | -1.35  (-43.00,40.29) | -0.93  (-42.45,40.60) | 0.00  (-38.83,38.83) | NCG |  |  |  |  |  |  |  |  |  |  |  |  |  |
| -28.00  (-73.34,17.35) | -18.53  (-45.75,8.68) | -18.92  (-52.32,14.47) | -15.80  (-52.55,20.95) | -18.00  (-64.09,28.10) | -14.65  (-51.80,22.50) | -11.00  (-44.94,22.93) | -9.61  (-37.91,18.68) | -17.50  (-96.97,61.97) | -5.73  (-34.33,22.87) | -5.20  (-41.67,31.26) | -4.35  (-32.50,23.81) | -3.92  (-31.90,24.05) | -2.99  (-27.54,21.55) | -2.99  (-48.93,42.95) | ARA |  |  |  |  |  |  |  |  |  |  |  |  |
| -41.00  (-345.80,263.80) | -31.53  (-334.17,271.10) | -31.92  (-335.18,271.33) | -28.80  (-332.44,274.84) | -31.00  (-335.91,273.92) | -27.65  (-331.34,276.04) | -24.00  (-327.32,279.31) | -22.61  (-325.35,280.12) | -30.50  (-342.21,281.21) | -18.73  (-321.50,284.03) | -18.20  (-321.81,285.41) | -17.35  (-320.07,285.38) | -16.92  (-319.63,285.78) | -15.99  (-318.40,286.41) | -15.99  (-320.88,288.90) | -13.00  (-316.34,290.34) | SPBS |  |  |  |  |  |  |  |  |  |  |  |
| -30.04  (-72.64,12.55) | -20.58  (-42.85,1.70) | -20.97  (-50.51,8.57) | -17.84  (-51.15,15.46) | -20.04  (-63.44,23.35) | -16.69  (-48.48,15.10) | -13.05  (-43.21,17.11) | -11.66  (-35.26,11.95) | -19.54  (-97.48,58.39) | -7.78  (-31.76,16.20) | -7.25  (-40.23,25.74) | -6.39  (-29.93,17.15) | -5.97  (-29.22,17.29) | -5.04  (-24.04,13.96) | -5.04  (-48.27,38.19) | -2.04  (-32.49,28.40) | 10.96  (-291.99,313.90) | AR |  |  |  |  |  |  |  |  |  |  |
| -31.70  (-71.42,8.03) | -22.23  (-38.52,-5.94) | -22.62  (-47.89,2.64) | -19.50  (-49.04,10.04) | -21.70  (-62.28,18.88) | -18.35  (-48.40,11.70) | -14.70  (-40.65,11.25) | -13.31  (-31.34,4.71) | -21.20  (-97.60,55.20) | -9.43  (-27.90,9.03) | -8.90  (-38.04,20.24) | -8.05  (-25.69,9.60) | -7.62  (-25.08,9.84) | -6.69  (-17.66,4.27) | -6.69  (-47.04,33.66) | -3.70  (-29.98,22.58) | 9.30  (-293.25,311.85) | -1.66  (-22.96,19.65) | EEN |  |  |  |  |  |  |  |  |  |
| -32.18  (-72.51,8.15) | -22.72  (-40.34,-5.09) | -23.11  (-49.29,3.07) | -19.98  (-50.33,10.37) | -22.18  (-63.35,18.99) | -18.83  (-45.15,7.48) | -15.19  (-42.05,11.67) | -13.80  (-33.05,5.46) | -21.68  (-98.40,55.04) | -9.92  (-29.62,9.78) | -9.39  (-39.39,20.61) | -8.53  (-27.59,10.53) | -8.11  (-26.89,10.68) | -7.18  (-20.33,5.97) | -7.18  (-48.18,33.82) | -4.18  (-31.37,23.00) | 8.82  (-293.82,311.45) | -2.14  (-22.61,18.33) | -0.48  (-16.66,15.69) | Coffee |  |  |  |  |  |  |  |  |
| -33.01  (-78.08,12.06) | -23.55  (-50.30,3.21) | -23.94  (-56.96,9.08) | -20.82  (-57.23,15.60) | -23.01  (-68.84,22.81) | -19.66  (-56.48,17.15) | -16.02  (-49.58,17.54) | -14.63  (-42.48,13.23) | -22.52  (-101.83,56.80) | -10.75  (-38.91,17.41) | -10.22  (-46.34,25.91) | -9.36  (-37.07,18.35) | -8.94  (-36.47,18.59) | -8.01  (-32.05,16.03) | -8.01  (-53.68,37.66) | -5.02  (-38.84,28.80) | 7.98  (-295.32,311.28) | -2.97  (-33.01,27.07) | -1.32  (-27.12,24.49) | -0.83  (-27.56,25.89) | AR+EEN |  |  |  |  |  |  |  |
| -41.00  (-112.25,30.25) | -31.53  (-92.86,29.80) | -31.92  (-96.23,32.38) | -28.80  (-94.92,37.32) | -31.00  (-102.73,40.73) | -27.65  (-93.99,38.69) | -24.00  (-88.59,40.58) | -22.61  (-84.43,39.20) | -30.50  (-127.12,66.12) | -18.73  (-80.69,43.22) | -18.20  (-84.16,47.75) | -17.35  (-79.10,44.40) | -16.92  (-78.59,44.75) | -15.99  (-76.19,44.20) | -15.99  (-87.63,55.64) | -13.00  (-77.72,51.72) | -0.00  (-308.28,308.28) | -10.96  (-73.79,51.87) | -9.30  (-70.22,51.62) | -8.82  (-70.13,52.50) | -7.98  (-72.52,56.55) | SAB |  |  |  |  |  |  |
| -36.19  (-81.54,9.16) | -26.73  (-53.95,0.50) | -27.12  (-60.52,6.28) | -23.99  (-60.75,12.77) | -26.19  (-72.29,19.91) | -22.84  (-60.00,14.32) | -19.20  (-53.14,14.74) | -17.81  (-46.11,10.50) | -25.69  (-105.17,53.78) | -13.93  (-42.54,14.68) | -13.39  (-49.87,23.08) | -12.54  (-40.70,15.62) | -12.12  (-40.10,15.87) | -11.19  (-35.75,13.37) | -11.19  (-57.13,34.76) | -8.19  (-42.39,26.00) | 4.81  (-298.54,308.15) | -6.15  (-36.61,24.31) | -4.49  (-30.78,21.80) | -4.01  (-31.20,23.18) | -3.18  (-37.01,30.65) | 4.81  (-59.92,69.53) | IIB |  |  |  |  |  |
| -38.25  (-79.22,2.73) | -28.78  (-47.85,-9.71) | -29.17  (-55.18,-3.17) | -26.05  (-57.25,5.15) | -28.25  (-70.05,13.56) | -24.90  (-56.57,6.77) | -21.26  (-49.08,6.57) | -19.86  (-37.20,-2.53) | -27.75  (-104.81,49.31) | -15.98  (-36.98,5.01) | -15.45  (-46.32,15.41) | -14.60  (-34.98,5.79) | -14.17  (-34.31,5.97) | -13.25  (-28.26,1.77) | -13.25  (-54.88,28.39) | -10.25  (-38.39,17.88) | 2.75  (-299.97,305.47) | -8.21  (-31.66,15.25) | -6.55  (-24.26,11.16) | -6.07  (-25.09,12.96) | -5.23  (-32.93,22.46) | 2.75  (-58.99,64.49) | -2.06  (-30.20,26.09) | ACU |  |  |  |  |
| -41.08  (-88.42,6.26) | -31.61  (-62.03,-1.20) | -32.01  (-68.06,4.05) | -28.88  (-68.07,10.31) | -31.08  (-79.14,16.98) | -27.73  (-63.88,8.42) | -24.09  (-60.64,12.47) | -22.69  (-54.08,8.70) | -30.58  (-111.21,50.04) | -18.82  (-50.48,12.85) | -18.28  (-57.20,20.63) | -17.43  (-48.70,13.84) | -17.00  (-48.11,14.10) | -16.08  (-44.14,11.99) | -16.08  (-63.99,31.83) | -13.08  (-49.87,23.71) | -0.08  (-303.73,303.56) | -11.04  (-43.19,21.11) | -9.38  (-38.98,20.22) | -8.90  (-33.69,15.89) | -8.07  (-44.52,28.39) | -0.08  (-66.22,66.05) | -4.89  (-41.69,31.91) | -2.83  (-34.08,28.42) | Tea |  |  |  |
| -40.01  (-81.31,1.29) | -30.55  (-50.29,-10.81) | -30.94  (-58.59,-3.29) | -27.81  (-59.44,3.82) | -30.01  (-72.13,12.11) | -26.66  (-52.69,-0.64) | -23.02  (-51.32,5.28) | -21.63  (-42.83,-0.42) | -29.51  (-106.75,47.72) | -17.75  (-39.36,3.87) | -17.21  (-48.51,14.08) | -16.36  (-37.40,4.68) | -15.94  (-36.72,4.85) | -15.01  (-30.89,0.87) | -15.01  (-56.96,26.94) | -12.01  (-40.62,16.59) | 0.99  (-301.78,303.75) | -9.97  (-30.13,10.19) | -8.31  (-26.79,10.16) | -7.83  (-21.00,5.34) | -7.00  (-35.17,21.17) | 0.99  (-60.97,62.94) | -3.82  (-32.43,24.79) | -1.76  (-22.77,19.24) | -1.07  (-29.14,27.00) | Water |  |  |
| -41.00  (-79.36,-2.64) | -31.53  (-44.04,-19.03) | -31.92  (-54.96,-8.88) | -28.80  (-56.48,-1.12) | -31.00  (-70.25,8.25) | -27.65  (-55.86,0.56) | -24.00  (-47.82,-0.19) | -22.61  (-37.32,-7.91) | -30.50  (-106.20,45.20) | -18.73  (-34.02,-3.45) | -18.20  (-45.50,9.10) | -17.35  (-31.78,-2.92) | -16.92  (-31.00,-2.84) | -15.99  (-20.27,-11.72) | -15.99  (-55.06,23.07) | -13.00  (-37.17,11.17) | -0.00  (-302.38,302.38) | -10.96  (-29.47,7.56) | -9.30  (-19.62,1.01) | -8.82  (-21.25,3.62) | -7.98  (-31.64,15.67) | -0.00  (-60.04,60.04) | -4.81  (-28.99,19.38) | -2.75  (-17.15,11.65) | -0.08  (-27.82,27.65) | -0.99  (-16.28,14.31) | Control |  |
| -67.70  (-127.98,-7.42) | -58.23  (-106.41,-10.06) | -58.62  (-110.52,-6.72) | -55.50  (-109.61,-1.38) | -57.70  (-118.54,3.15) | -54.35  (-108.74,0.05) | -50.70  (-102.94,1.54) | -49.31  (-98.10,-0.52) | -57.20  (-146.04,31.64) | -45.43  (-94.39,3.52) | -44.90  (-98.80,9.00) | -44.04  (-92.70,4.61) | -43.62  (-92.21,4.96) | -42.69  (-89.34,3.95) | -42.69  (-103.39,18.00) | -39.70  (-92.10,12.71) | -26.70  (-332.63,279.23) | -37.65  (-87.75,12.44) | -36.00  (-81.34,9.34) | -35.51  (-83.65,12.62) | -34.68  (-86.85,17.49) | -26.70  (-102.64,49.24) | -31.51  (-83.92,20.90) | -29.45  (-78.12,19.23) | 26.62  (-27.53,80.76) | -27.69  (-76.64,21.27) | -26.70  (-73.20,19.80) | EPN |

S5 Table of pairwise comparisons of the normal eating time (MD, 95% CI)

| AR | OA-H |  | EEN | EACU+CG | EEN+GPA | OA-L | Coffee | IIB | SPBS | CG | EACU | Water | EA | ARA | LAX | IA | GPA-H | SAB | ANT | GPA-L | THR | Control | ACU |
| --- | --- | --- | --- | --- | --- | --- | --- | --- | --- | --- | --- | --- | --- | --- | --- | --- | --- | --- | --- | --- | --- | --- | --- |
| AR |  |  |  |  |  |  |  |  |  |  |  |  |  |  |  |  |  |  |  |  |  |  |  |
| -10.00  (-58.57,38.57) | OA-H |  |  |  |  |  |  |  |  |  |  |  |  |  |  |  |  |  |  |  |  |  |  |
| -14.87  (-52.28,22.54) | -4.87  (-43.76,34.02) |  | EEN |  |  |  |  |  |  |  |  |  |  |  |  |  |  |  |  |  |  |  |  |
| -16.67  (-62.25,28.90) | -6.67  (-53.47,40.12) |  | -1.80  (-36.84,33.23) | EACU+CG | |  |  |  |  |  |  |  |  |  |  |  |  |  |  |  |  |  |  |
| -18.14  (-62.97,26.69) | -8.14  (-54.21,37.93) |  | -3.27  (-33.06,26.52) | -1.47  (-44.36,41.42) | EEN+GPA |  |  |  |  |  |  |  |  |  |  |  |  |  |  |  |  |  |  |
| -20.00  (-69.06,29.06) | -10.00  (-43.98,23.98) |  | -5.13  (-44.62,34.36) | -3.33  (-50.63,43.97) | -1.86  (-48.45,44.72) | OA-L |  |  |  |  |  |  |  |  |  |  |  |  |  |  |  |  |  |
| -24.61  (-64.30,15.09) | -14.61  (-55.69,26.48) |  | -9.74  (-36.45,16.98) | -7.93  (-45.42,29.56) | -6.47  (-42.96,30.03) | -4.61  (-46.27,37.06) | Coffee |  |  |  |  |  |  |  |  |  |  |  |  |  |  |  |  |
| -28.84  (-76.82,19.15) | -18.84  (-67.98,30.31) |  | -13.97  (-52.12,24.18) | -12.17  (-58.35,34.02) | -10.70  (-56.16,34.76) | -8.84  (-58.46,40.79) | -4.23  (-44.63,36.16) | IIB |  |  |  |  |  |  |  |  |  |  |  |  |  |  |  |
| -19.00  (-336.84,298.84) | -9.00  (-327.02,309.02) |  | -4.13  (-320.64,312.37) | -2.33  (-319.90,315.24) | -0.86  (-318.33,316.61) | 1.00  (-317.10,319.09) | 5.60  (-311.18,322.39) | 9.84  (-308.09,327.77) | SPBS |  |  |  |  |  |  |  |  |  |  |  |  |  |  |
| -30.79  (-66.50,4.93) | -20.79  (-58.04,16.47) |  | -15.92  (-35.28,3.44) | -14.11  (-47.37,19.14) | -12.65  (-44.46,19.17) | -10.79  (-48.67,27.10) | -6.18  (-30.73,18.36) | -1.95  (-38.44,34.54) | -11.78  (-328.09,304.53) | CG |  |  |  |  |  |  |  |  |  |  |  |  |  |
| -31.24  (-70.06,7.58) | -21.24  (-61.48,19.00) |  | -16.37  (-41.95,9.20) | -14.57  (-45.47,16.33) | -13.10  (-48.70,22.50) | -11.24  (-52.07,29.59) | -6.64  (-35.52,22.25) | -2.40  (-41.94,37.13) | -12.24  (-328.91,304.44) | -0.45  (-23.58,22.67) | EACU |  |  |  |  |  |  |  |  |  |  |  |  |
| -31.20  (-83.86,21.46) | -21.20  (-74.92,32.52) |  | -16.33  (-60.05,27.38) | -14.53  (-65.55,36.49) | -13.06  (-63.35,37.23) | -11.20  (-65.36,42.96) | -6.60  (-41.20,28.01) | -2.36  (-55.55,50.83) | -12.20  (-330.87,306.47) | -0.42  (-42.84,42.01) | 0.04  (-45.04,45.11) | Water |  |  |  |  |  |  |  |  |  |  |  |
| -42.66  (-218.36,133.04) | -32.66  (-208.69,143.36) |  | -27.79  (-201.05,145.47) | -25.99  (-201.21,149.23) | -24.52  (-199.54,150.50) | -22.66  (-198.82,153.50) | -18.06  (-191.84,155.73) | -13.82  (-189.69,162.04) | -23.66  (-383.72,336.41) | -11.87  (-184.79,161.04) | -11.42  (-185.01,162.17) | -11.46  (-188.65,165.74) | EA |  |  |  |  |  |  |  |  |  |  |
| -34.79  (-75.70,6.12) | -24.79  (-67.05,17.47) |  | -19.92  (-48.67,8.82) | -18.12  (-56.90,20.66) | -16.65  (-54.56,21.26) | -14.79  (-57.61,28.03) | -10.18  (-41.85,21.48) | -5.95  (-47.54,35.63) | -15.79  (-332.72,301.15) | -4.00  (-30.50,22.49) | -3.55  (-34.10,27.01) | -3.59  (-50.49,43.31) | 7.87  (-166.19,181.94) | ARA |  |  |  |  |  |  |  |  |  |
| -40.60  (-93.65,12.45) | -30.60  (-84.71,23.50) |  | -25.73  (-70.09,18.62) | -23.93  (-75.36,27.50) | -22.46  (-73.24,28.31) | -20.60  (-75.14,33.94) | -16.00  (-62.30,30.30) | -11.76  (-65.34,41.81) | -21.60  (-340.33,297.13) | -9.82  (-52.75,33.12) | -9.36  (-54.91,36.19) | -9.40  (-67.20,48.40) | 2.06  (-175.26,179.37) | -5.81  (-53.15,41.53) | LAX |  |  |  |  |  |  |  |  |
| -59.45  (-239.05,120.14) | -49.45  (-229.36,130.45) |  | -44.59  (-221.79,132.62) | -42.78  (-221.90,136.34) | -41.31  (-220.24,137.61) | -39.45  (-219.49,140.59) | -34.85  (-212.57,142.87) | -30.62  (-210.37,149.14) | -40.45  (-402.43,321.53) | -28.67  (-205.54,148.20) | -28.21  (-205.74,149.31) | -28.25  (-209.31,152.80) | -16.79  (-54.12,20.53) | -24.66  (-202.66,153.33) | -18.85  (-200.02,162.32) | IA |  |  |  |  |  |  |  |
| -41.34  (-85.74,3.06) | -31.34  (-76.99,14.31) |  | -26.47  (-60.03,7.09) | -24.67  (-67.12,17.79) | -23.20  (-64.87,18.46) | -21.34  (-67.51,24.83) | -16.74  (-52.81,19.34) | -12.50  (-57.53,32.53) | -22.34  (-339.75,295.07) | -10.55  (-42.19,21.08) | -10.10  (-45.20,25.00) | -10.14  (-60.13,39.85) | 1.32  (-173.60,176.24) | -6.55  (-43.95,30.84) | -0.74  (-51.13,49.66) | 18.11  (-160.72,196.94) | GPA-H |  |  |  |  |  |  |
| -67.00  (-253.93,119.92) | -57.00  (-244.23,130.23) |  | -52.13  (-236.78,132.51) | -50.33  (-236.81,136.14) | -48.86  (-235.16,137.43) | -47.00  (-234.36,140.35) | -42.40  (-227.52,142.73) | -38.16  (-225.24,148.91) | -48.00  (-413.68,317.67) | -36.22  (-220.53,148.10) | -35.76  (-220.70,149.18) | -35.80  (-224.13,152.53) | -24.34  (-276.47,227.78) | -32.21  (-217.60,153.17) | -26.40  (-214.84,162.04) | -7.55  (-262.40,247.30) | -25.66  (-211.85,160.53) | SAB |  |  |  |  |  |
| -41.95  (-88.58,4.68) | -31.95  (-79.77,15.87) |  | -27.08  (-63.21,9.04) | -25.28  (-70.05,19.49) | -23.81  (-67.71,20.08) | -21.95  (-70.27,26.36) | -17.35  (-56.09,21.40) | -13.11  (-60.34,34.11) | -22.95  (-340.68,294.78) | -11.16  (-45.81,23.48) | -10.71  (-48.57,27.15) | -10.75  (-62.70,41.20) | 0.71  (-174.79,176.21) | -7.16  (-47.17,32.85) | -1.35  (-53.71,51.02) | 17.50  (-161.89,196.90) | -0.61  (-44.20,42.98) | 25.05  (-161.68,211.79) | ANT |  |  |  |  |
| -41.79  (-88.18,4.60) | -31.79  (-79.38,15.80) |  | -26.92  (-63.06,9.22) | -25.12  (-69.65,19.42) | -23.65  (-67.42,20.13) | -21.79  (-69.88,26.30) | -17.18  (-55.68,21.31) | -12.95  (-59.94,34.04) | -22.79  (-340.48,294.91) | -11.00  (-45.37,23.37) | -10.55  (-48.13,27.04) | -10.59  (-62.35,41.17) | 0.87  (-174.56,176.31) | -7.00  (-46.73,32.74) | -1.19  (-53.34,50.97) | 17.67  (-161.67,197.00) | -0.45  (-33.03,32.13) | 25.22  (-161.46,211.89) | 0.16  (-45.45,45.77) | GPA-L |  |  |  |
| -43.83  (-90.60,2.94) | -33.83  (-81.79,14.13) |  | -28.96  (-65.52,7.60) | -27.16  (-72.08,17.77) | -25.69  (-69.84,18.46) | -23.83  (-72.29,24.62) | -19.23  (-58.17,19.72) | -14.99  (-62.36,32.38) | -24.83  (-342.58,292.92) | -13.04  (-47.92,21.83) | -12.59  (-50.64,25.46) | -12.63  (-64.72,39.47) | -1.17  (-176.71,174.37) | -9.04  (-49.22,31.14) | -3.23  (-55.72,49.27) | 15.62  (-163.81,195.05) | -2.49  (-46.23,41.25) | 23.17  (-163.60,209.94) | -1.88  (-47.87,44.11) | -2.04  (-47.80,43.71) | THR |  |  |
| -43.00  (-76.52,-9.49) | -33.00  (-68.16,2.15) |  | -28.13  (-44.76,-11.51) | -26.33  (-57.22,4.56) | -24.86  (-54.65,4.92) | -23.00  (-58.83,12.82) | -18.40  (-39.67,2.87) | -14.16  (-48.51,20.18) | -24.00  (-340.07,292.07) | -12.22  (-24.55,0.12) | -11.76  (-31.35,7.82) | -11.80  (-52.42,28.82) | -0.34  (-172.82,172.14) | -8.21  (-31.67,15.24) | -2.40  (-43.52,38.72) | 16.45  (-159.99,192.89) | -1.66  (-30.79,27.46) | 24.00  (-159.90,207.90) | -1.05  (-33.47,31.37) | -1.22  (-33.29,30.86) | 0.83  (-31.80,33.45) | Control |  |
| -63.21  (-107.67,-18.76) | -53.21  (-98.92,-7.51) |  | -48.34  (-82.12,-14.57) | -46.54  (-86.09,-6.99) | -45.07  (-86.85,-3.29) | -43.21  (-89.43,3.01) | -38.61  (-74.79,-2.43) | -34.37  (-79.46,10.71) | -44.21  (-361.63,273.21) | -32.43  (-64.16,-0.70) | -31.97  (-59.36,-4.58) | -32.01  (-82.07,18.05) | -20.55  (-195.49,154.38) | -28.42  (-65.88,9.03) | -22.61  (-73.05,27.83) | -3.76  (-182.60,175.08) | -21.87  (-63.11,19.37) | 3.79  (-182.41,189.99) | -21.26  (-64.92,22.40) | -21.42  (-64.80,21.95) | -19.38  (-63.18,24.41) | -20.21  (-49.41,9.00) | ACU |
